# Valorization of lignocellulosic biomass into sustainable materials for adsorption and photocatalytic applications in water and air remediation

**DOI:** 10.1007/s11356-023-27484-2

**Published:** 2023-05-25

**Authors:** Meriem Mergbi, Melissa Greta Galloni, Dominic Aboagye, Ehiaghe Elimian, Peidong Su, Belhadj M. Ikram, Walid Nabgan, Jorge Bedia, Hedi Ben Amor, Sandra Contreras, Francisco Medina, Ridha Djellabi‬‬‬‬‬‬‬‬

**Affiliations:** 1 Faculty of Sciences of Gabes, RL Processes, Energetic, Environment and Electric Systems (PEESE), University of Gabes, 6072 Gabes, Tunisia; 2 Department of Chemical Engineering, Universitat Rovira I Virgili, 43007 Tarragona, Spain; 3 Dipartimento di Chimica, Università Degli Studi Di Milano, Via Golgi 19, 20133 Milano, Italy; 4 Department of Chemical and Environmental Engineering, Faculty of Science and Engineering, University of Nottingham Ningbo China, Ningbo, 315100 China; 5 Department of Plant Biology and Biotechnology, Faculty of Life Sciences, University of Benin, PMB 1154, Benin City, Nigeria; 6 School of Chemical & Environmental Engineering, China University of Mining & Technology (Beijing), Beijing, 100083 China; 7 Department of Chemical and Environmental Engineering, Malaysia Japan International Institute of Technology, Universiti Teknologi Malaysia, Jalan Sultan Yahya Petra, 54100 Kuala Lumpur, Malaysia; 8 Chemical Engineering Department, Autonomous University of Madrid, Madrid, Spain

**Keywords:** Lignocellulosic biomass, Adsorption, Photocatalysis, Environmental remediation, Circular bioeconomy

## Abstract

An exponential rise in global pollution and industrialization has led to significant economic and environmental problems due to the insufficient application of green technology for the chemical industry and energy production. Nowadays, the scientific and environmental/industrial communities push to apply new sustainable ways and/or materials for energy/environmental applications through the so-called circular (bio)economy. One of today’s hottest topics is primarily valorizing available lignocellulosic biomass wastes into valuable materials for energy or environmentally related applications. This review aims to discuss, from both the chemistry and mechanistic points of view, the recent finding reported on the valorization of biomass wastes into valuable carbon materials. The sorption mechanisms using carbon materials prepared from biomass wastes by emphasizing the relationship between the synthesis route or/and surface modification and the retention performance were discussed towards the removal of organic and heavy metal pollutants from water or air (NO_x_, CO_2_, VOCs, SO_2_, and Hg^0^). Photocatalytic nanoparticle–coated biomass-based carbon materials have proved to be successful composites for water remediation. The review discusses and simplifies the most raised interfacial, photonic, and physical mechanisms that might take place on the surface of these composites under light irradiation. Finally, the review examines the economic benefits and circular bioeconomy and the challenges of transferring this technology to more comprehensive applications.

## Introduction 

Production process through the linear economy for many decades has led to serious environmental, economic, and ecosystem perturbations (Jørgensen et al. 2018; Turner 2018). Linear economy generates huge quantities of waste in the aquatic and atmospheric environment. Hence, the scientific and industrial communities worldwide actively search for green approaches to decrease the quantity and hazardous of generated waste and their possible recycling (Nabgan et al. 2023). The concept of “zero waste theory” is based on using green and recyclable products and valorizing natural and available resources (Romano et al. 2019). As a result, the bioeconomy European Union must include sustainability and circularity at the core of its industrial makeup to effectively transition from a linear to a circular economy. By 2060, international energy agencies anticipated that the world’s need for bioenergy would multiplied (Brahma et al. 2022), suggesting a need to switch to renewable feedstock for heat generation, chemicals, fuels, and electricity, such as lignocellulosic biomass, is gaining more and more attention (Djellabi et al. 2022). Lignocellulosic biomass is a carbon-rich material that can be employed in the sustainable route for bioenergy production and high-value products as well as multifunctional materials for environmental remediation (Bhatnagar et al. 2015; Nouacer et al. 2015; Khelaifia et al. 2016; Wang et al. 2021).

The production of carbon materials from lignocellulosic biomass wastes has gained attention in recent years, particularly due to its low cost and relative earth abundance (Ziati et al. 2012; Yahya et al. 2015; Ikram et al. 2022). Lignocellulosic biomass mainly contains three components: lignin, cellulose, and hemicellulose, wherein lignin is the central part. Lignin is rich in carbon atoms compared to the other components, and therefore, it is regarded as the best precursor to produce activated carbon (Ikram et al. 2022). The synthesis of high-quality activated carbon significantly depends on different factors, such as the starting carbonaceous precursor, carbonization and activation approaches, and other synthesis conditions (Carrott and Carrott 2007). In fact, an extensive list of biomass wastes was converted into activated carbon, such as date stones (Abderrahim et al. 2022a), olive pits (Redondo et al. 2015), coconut shells (Gratuito et al. 2008), lignin (Carrott and Carrott 2007), barley straw (Pallarés et al. 2018), orange peels (Köseoğlu and Akmil-Başar 2015), soybean oil cake (Tay et al. 2009), tomato (Sayğılı and Güzel 2016), chestnut shell (Duan et al. 2021), wood (Danish and Ahmad 2018), palm shell (Hamada et al. 2020), and walnut shell (Plaza et al. 2010). Different approaches are used for the activation to ensure effective carbonization, including the chemical, physical, and physicochemical ones. In terms of chemical activation, several agents can be used, such as KOH (Khalil et al. 2013), FeCl_3_ (Bedia et al. 2020), ZnCl_2_ (Caturla et al. 1991), H_3_PO_4_ (Yorgun and Yıldız 2015), NaOH (Islam et al. 2017), K_2_CO_3_ (Sayğılı and Sayğılı 2019), and K_2_B_4_O_7_ (Gao et al. 2015). In addition, the synthesis method significantly affects several features, such as the surface area, internal porosity, oxygenated functional groups, and physical properties (González-García 2018). Different approaches were reported to assist the activation and carbonization, such as hydrothermal (Hoekman et al. 2011), microwave (Paramasivan 2022), ultrasonic (Xu et al. 2019), and vacuum (Yousaf et al. 2021).

Lignocellulosic biomass has been extensively used as support for photocatalytic nanoparticles to improve the pollutant removal efficiency during environmental remediation. TiO_2_/carbon composites are some of the successful photocatalytic materials for water purification because of the synergistic effects in terms of enhanced light absorption, excellent adsorptive ability, and promoted charges transferred (Puma et al. 2008; Lisowski et al. 2018; Djellabi et al. 2019a, b, c, d; Djellabi et al. 2019a, b, c, d; Djellabi et al. 2021a, b, c). Several photocatalytic mechanistic pathways have been reported regarding oxidation and reduction of pollutants on the surface of titania-carbon materials (Matos et al. 2010; Asiltürk and Şener 2012; Djellabi et al. 2019a, b, c, d). Besides, a wide list of photocatalysts has been supported on the surface of lignocellulosic biomass or lignocellulosic biomass–based activated carbon such as ZnO (Raizada et al. 2014), Ag_3_PO_4_ (Abderrahim et al. 2022b), CuO (Khaled et al. 2020), g-C_3_N_4_ (Pi et al. 2015), and MoS_2_ (Ye et al. 2019).

The present review discusses the valorization of biomass from both fundamental and applied research. It includes most of recent data on the synthesis of carbon based materials to be used for environmental application. Differently to previous reported reviews, we stressed in this review the mechanistic pathways that take place during the synthesis of carbon materials from biomass wastes. In addition, it provides the most a summary regarding the mechanistic pathways taking place for the removal of materials through simple adsorption or photocatalytic route from air and water. The adsorption mechanisms towards the removal of organic pollutants, heavy metals, CO_2_, VOCs, NO_x_, and mercury are systematically reviewed. In-depth discussion was held on the photocatalytic behavior of semiconductor-supported lignocellulosic biomass. The most important uses of valorized lignocellulosic biomass–based materials for adsorption and photocatalytic removal of pollutants from the aquatic and atmospheric environment are outlined in the paper.

## Synthesis of carbon from lignocellulose biomass

**Fig. 1:**
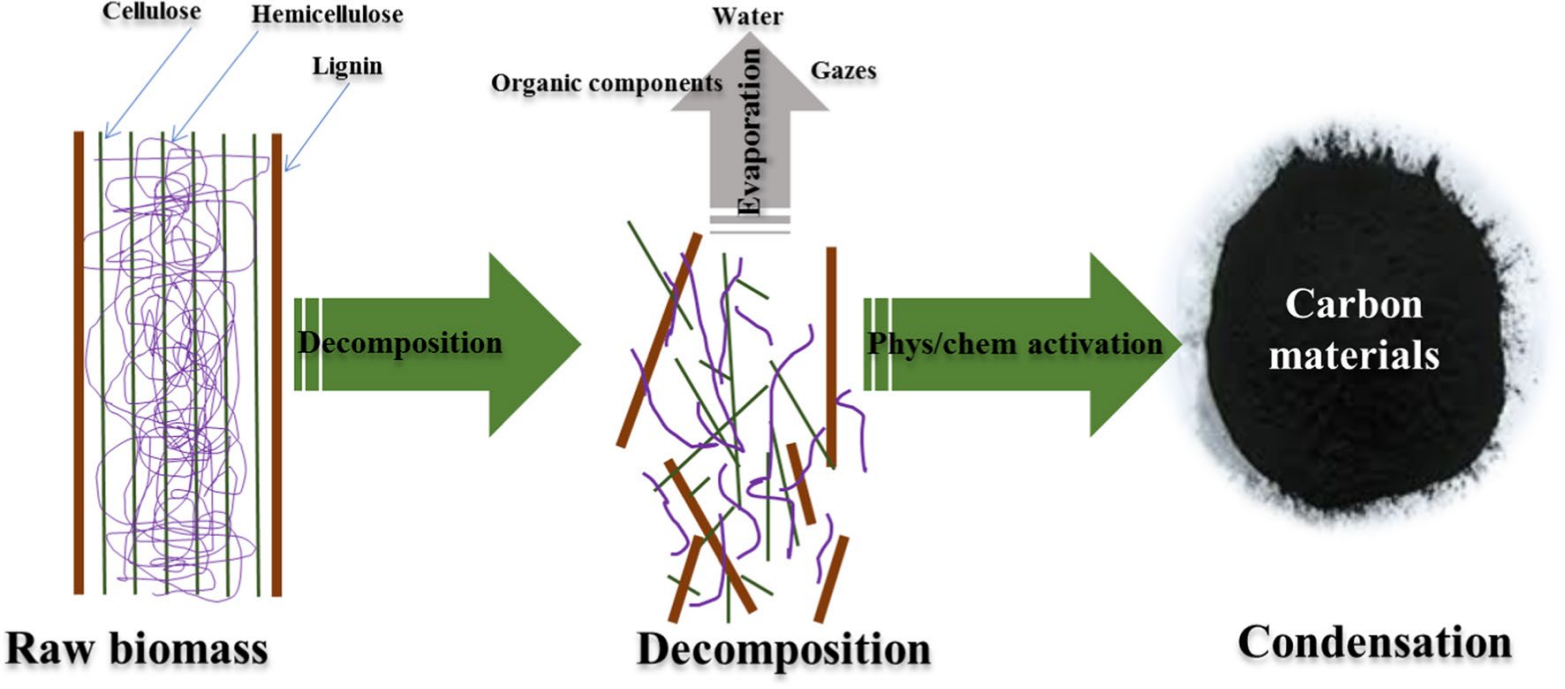
Main steps towards the conversion of biomass into activated carbon materials

Due to the urgent demand for materials for environmental remediation and the high cost of available commercial materials, a lot of studies have been reported by the scientific community to find sustainable and low-cost alternative routes to fabricate highly adsorptive or functionalized carbons. The valorization of lignocellulosic biomass–based agricultural or industrial wastes has been a hot topic over the last few years. Lignocellulosic biomass contains cellulose, hemicellulose, and lignin. After cellulose, lignin is considered an abundant carbon source material, annually producing 40–50 million tons (Carrott and Carrott 2007). The use of lignocellulosic biomass as a precursor for producing carbon materials is a successful option because of the rich carbon content in lignin. Numerous sources of lignocellulosic biomass can be used to produce activated carbon with a high adsorption capacity, such as durian shells (Chandra et al. 2007), coconut shells (Li et al. 2008), and food waste (Kosheleva et al. 2019). Palm kernel shell (Sumathi et al. 2009), rice husk (Dahlan et al. 2006; Heo and Park 2015), pistachio-nut shell (Yang and Lua 2003), olive pits (Djellabi et al. 2019a, b, c, d), date stones (Khelaifia et al. 2016), palm tree (Nouacer et al. 2015), and wood (Danish and Ahmad 2018, Musa et al. 2019). Activated carbon derived from lignocellulosic biomass is used in a broad range of applications for both industrial and residential uses, including wastewater treatment (Thompson et al. 2016; Wong et al. 2018), air pollution treatment(Devinny et al. 2017), petroleum refining (Cagnon et al. 2009), storage (Zieliński et al. 2005), and catalysis (Tsai et al. 1998). The advantages of activated carbon materials are their stability, high surface area up to 3000 m^2^/g, and porosity (El Hadrami et al. 2022). In general, biomass conversion into carbon materials passes through two steps, decomposition and evaporation followed by carbon condensation (Fig. 1). In this section, the most common synthesis approaches of chars and activated carbons from biomass were summarized and discussed.

### Carbonization

Carbonization, also known as pyrolysis, is a process used to raise the carbon yield of a carbonaceous material by eliminating non-carbon elements under high temperatures resulting in an observable increase in the initial porosity obtained char before the subsequent improvement during the activation phase. Since the carbonization process has a significant impact on the final product, it is crucial to select the parameters carefully. The most significant effects on carbonization are the temperature range and heating rate, with residence time and inert gas flow rate also having a significant impact (Wang et al. 2020a, b, c, d, e, f).

**Fig. 2:**
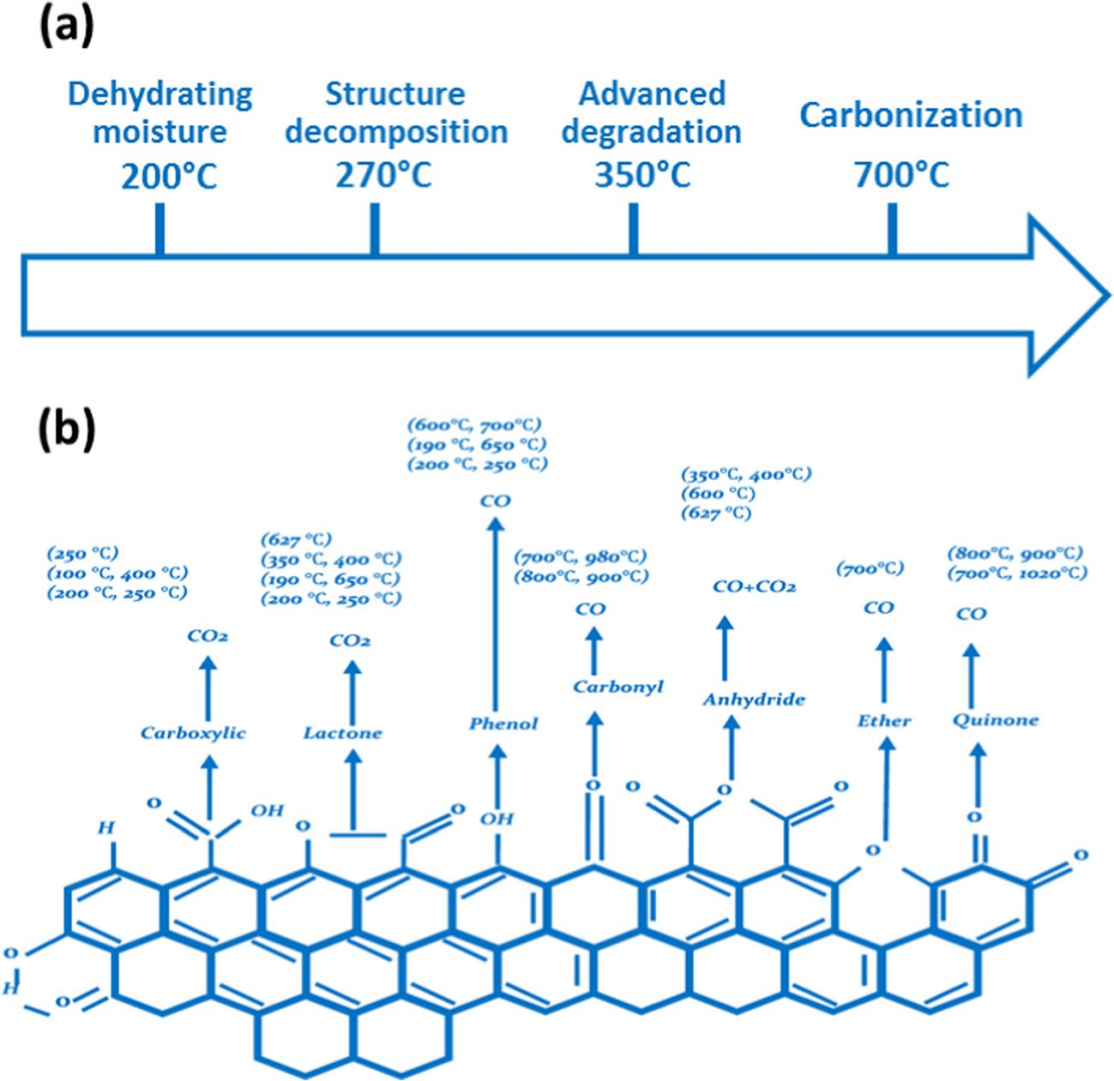
**a** Main steps of biomass pyrolysis to produce activated carbon. **b** Decomposition of oxygen functional groups on the surface of biomass materials as a function of heat treatment. Reproduced with permission from Shafeeyan et al. (2010); copyright 2023 Elsevier

To avoid biomass oxidation and to minimize the emission of volatile gases and water during the carbonization process, an oxygen-free environment must be maintained as much as is practicable. As shown in Fig. 2a, pyrolysis involves four stages: (i) dehydrating moisture from biomass at temperatures below 200 °C; (ii) decomposition of biomass structure to release volatiles and organic acids at temperatures between 170 and 270 °C; (iii) advanced decomposition at 270–350 °C to release liquids and gas, ending up with biochar generation; and (iv) removal of organic components to form activated carbon at temperature up to 700 °C (Ikram et al. 2022). It is challenging to create a perfectly inert environment for continuous pyrolysis since oxygen is present in the vacuum area of bulk biomass (Lua et al. 2006). Therefore, to evaluate product yields and quality, it is crucial to understand how oxygen impacts biomass pyrolysis, as a study has reported that the presence of oxygen during pyrolysis contributes to an increase in both the specific heat capacity and total pore volume (Zhang et al. 2016a, b). Also, the treatment of lignocellulosic biomass at high-temperature values can destroy many oxygen functional groups that in turn change the surface chemistry of the produced carbon. Figure 2 b shows that the decomposition of oxygen functional groups occurs at different calcination temperature values.

Based on the heating rate, pyrolysis can be divided into classes: slow, fast, flash, and intermediate (Laird et al. 2009). At higher temperatures, the yield of biochar is reduced as it has a higher carbon content as a consequence of the deeper devolatilization of the biomass precursor (Colantoni et al. 2016). In general, to control the biochar yield, low heating rates (from 10 to 15 °C/min) are applied along with a medium carbonization temperature (Ioannidou and Zabaniotou 2007). The characteristics of biochar, such as the surface chemistry, pore volume, pore size distribution, and elemental composition, are all mainly influenced by the pyrolysis temperature (Ahmad et al. 2013; Shen et al. 2017).

### Torrefaction

Torrefaction is a mild pyrolysis technique used to convert the biomass’s amorphous and crystalline regions to hydrophobic products with high energy density (Rousset et al. 2012). The principal advantages of this biomass pre-treatment are as follows: (i) reduced transportation costs, (ii) low grinding energy, (iii) low milling cost, (iv) cheap chemical treatment and low waste generation, and (v) rapid reactivity, besides to an east to scale and non-corrosive process (Nunes et al. 2014; Acharya et al. 2015). Torrefied by-product yields and attributes depend on several factors, including the biomass type, particle size, processing temperature and time, heating rate, and working atmosphere composition. Much research has been done to determine how these operational factors affect the torrefaction (Shankar Tumuluru et al. 2011; Olugbade and Ojo 2020). It was observed that reducing the temperature at which raw materials are prepared increases the yields and enables the production of a more energetically dense material (Krysanova et al. 2019). The carbon content of the materials increases with the torrefaction time, increasing also the calorific value. Chen and Kuo (2010) concluded that the primary process of biomass decomposition takes place in the first hour and then begin to slow down. The size of the biomass particles is another critical factor in biomass torrefaction that influences the mechanism of process. Wang et al. reported that a greater particle size affected the reactor’s efficiency and decreased the energy yield of biochars. The biomass’s structure and composition have an impact on the torrefaction process as well. It was discovered that alkali metals, like potassium and sodium, present in the biomass accelerated its decomposition during the torrefaction (Wang et al. 2017). Particularly, De Macedo et al. claimed that potassium influences how biopolymers interact, reducing the torrefaction period and increasing the output of important chemicals like guaiacol (De Macedo et al. 2018). The structural content of the biomass determines the temperature range of torrefaction. It has been demonstrated that biomass with a high content of hemicelluloses is effectively treated with moderate torrefaction (between 200 and 235 °C). In contrast, materials with a high cellulose and lignin content benefit most from heavy torrefaction (between 235 and 275 °C) (Chen and Kuo 2010). Currently, two types of torrefaction are employed: dry and wet.

#### Dry torrefaction

In nature, the process of dry torrefaction is one of the thermochemical conversion processes. This process occurs in the presence of an inert gas at temperatures ranging from 200 to 300 °C for around 30 to 60 min. In this sense, only 10% biomass energy is lost as gases (Bergman et al. 2005). Consequently, the torrefied solid product’s specific energy density increases when the biomass is heated at about 50 °C *per* min. Dry torrefaction of biomass causes several reactions, such as decarboxylation, dehydration, decarbonylation, demethoxylation, intermolecular rearrangement, condensation, and aromatization chemicals (Chen and Kuo 2010; Acharya et al. 2015). Typically, torrefaction is the initial pyrolysis process; hence, the end product, which still contains some volatile organic components, is not referred to as “biochar.” Generally speaking, the physicochemical properties of torrefied biomass fall between those of raw biomass and those of biochar (Kambo and Dutta 2015).

#### Wet torrefaction

Wet torrefaction (WT) is known as hydrothermal torrefaction (HTT) or hydrothermal carbonization (HTC) is carried out in the presence of an inert gas and subcritical pressured water vessel under 2 to 10 MPa at temperatures between 180 and 265 °C for treatment times ranging from five minutes to several hours. (Mumme et al. 2011; Xiao et al. 2012). According to Akshay et al. subcritical water has good solubility property in liquid form because of its dielectric point and high density compared to its vapor form (Jain et al. 2016). Because of its higher ionization constant, water can hydrolyze organic molecules at high temperatures, and acids or bases can speed up this process. Due to the creation of different organic acids, including acetic, levulinic, and formic, during the hydrothermal carbonization process, a pH reduction is frequently observed. These organic acids help to accelerate the hydrolysis and fragmentation of oligomers and monomers into smaller pieces that have little effect on lignin. As a result, solid biochar is produced with lower equilibrium moisture content than raw biomass (Demir-Cakan et al. 2009). Table 1 summarizes the main conditions and factors in dry and wet torrefaction approaches.

**Table 1:** Summary of dry and wet torrefaction

Process parameters	Dry torrefaction	Wet torrefaction	Ref
Temperature (°C)	200–300	185–265	Hoekman et al. (2011), Koppejan et al. (2012)
Pressure (atm) Residence (min)	1 10–120	1–200 1–240	Kambo and Dutta (2015) Kambo and Dutta (2015)
Inert environment Pre-drying	Yes Yes	Yes No	Masoumi et al. (2021) Reza et al. (2013)
Capability of handling moisture Enhanced heating value	Low Lower	High Higher	Lynam et al. (2015), Masoumi et al. (2021) Acharya et al. (2015)
Solid mass yield Commercial application Capability for handling feedstock	Higher Pilot scale Minimum moistures	Lower –- High moistures	Acharya et al. (2015) Acharya et al. (2015) Acharya et al. (2015)

### Microwave-assisted carbonization

Microwave heating systems have recently gained acceptance among scientists as an alternative to conventional heating sources and reactors attributed mainly to various inherent advantages, such as quick heating, selective heating, high controllability, low thermal inertia, and good treatment effects (Hejazifar et al. 2011). The microwave heat production is achieved at low-energy electromagnetic waves, termed microwaves have wavelengths between 0.001 and 0.3 m and frequencies between 1000 and 300,000 MHz (Komarov 2012). Instruments for measuring microwaves in laboratories (and homes) almost exclusively use microwaves with the frequency of 2450 MHz (or 12.2 cm wavelength) (Li et al. 2016a, b). Like all the electromagnetic waves, microwaves move at the speed of light (300,000 km/s) and consist of two oscillating field’s perpendicular to one another: an electric field and a magnetic field. The only effect of microwave photons, which have a low energy of 0.03–0.00003 kcal/mol, is the kinetic molecular excitation.

**Fig. 3:**
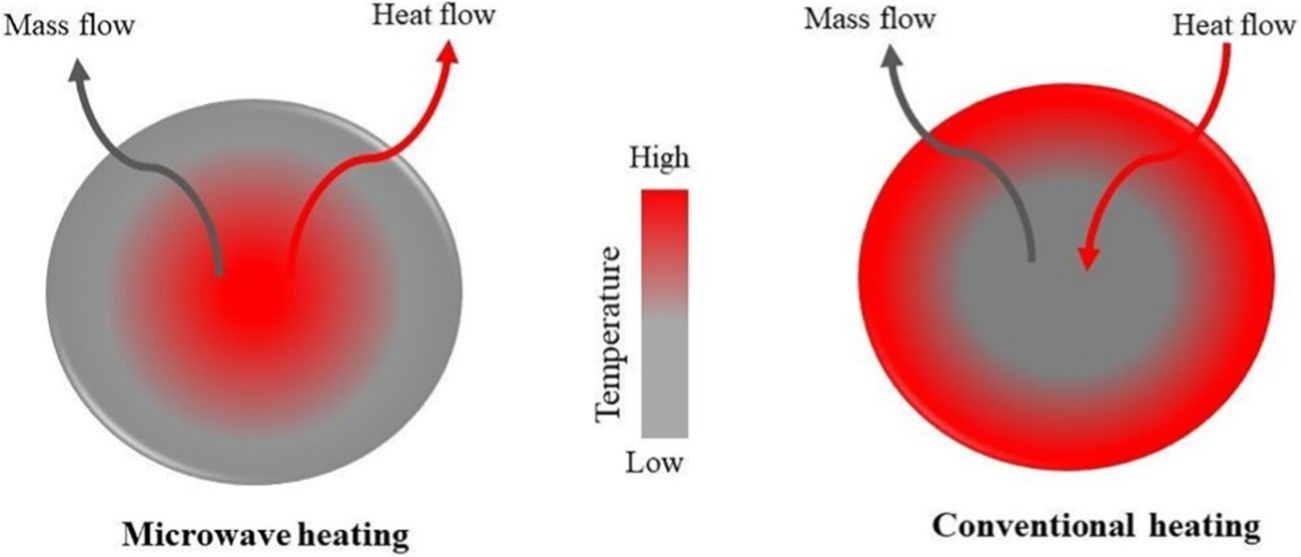
Mechanism of conductive heating and microwave heating (Omoriyekomwan et al. 2021); copyright 2023 Elsevier

With the help of these two fields, microwaves can produce heat. The electric field interacts with molecules in dipolar rotation and ionic conduction. In dipolar rotation, each rotating molecule pushes with the others to line up its dipole with the oscillating electric field. This friction generates heat (Li et al. 2016a, b). Energy transmission is more effective with microwaves than conventional heating methods because they directly interact with the components of a reaction mixture (Fig. 3) (Espinosa-Iglesias et al. 2015). Thermal conductivity underlies the conventional heating methods, involving the transfer of heat first from source to the vessel and then from vessel to the solution (Yang et al. 2010). The generated volatile matter moves from the particle’s interior at a high temperature to its surroundings with a lower temperature (An et al. 2020a, b). Thus, the thermal gradient problem with conventional heating techniques is resolved, improving the char yield. Biochar yield in microwave carbonization is primarily affected by the heating rate and radiation power (Ouyang et al. 2020). Table 2 summarizes the main advantages and disadvantages of the conventional and microwave-assisted carbonization.

**Table 2:** Advantages and disadvantages of conventional and microwave-assisted carbonization

Conventional heating		Microwave heating	
Advantages	Disadvantages	Advantages	Disadvantages
Simple process Easy scaling up Flexibility of feedstocks	Long carbonization Low-quality carbon materials Low textural uniformity Generation of high yield of toxic gases and by-products High applied temperature Consumption of high energy	Quick reaction High carbon materials quality High textural uniformity Low applied temperature Less used energy Facile pre-treatment Less production of toxic gas and by-products	Difficult scaling up Costly and complicated for the production of high amounts of carbon materials

### Activation

The purpose of the activation of biochar is to enhance the porosity and the surface area of the carbon materials, as well as the functionalization of the surface (Ahmed 2016). The activation procedure consists of these three basic steps: Firstly, since the elementary carbon crystal surface interact with the activation agents, viscous substances that clog pores must be removed, which is followed be the heating of the elementary carbon crystal and lastly the oxidation of the carbon species (Yang et al. 2010). Temperature and holding time significantly depends on the type of precursor, binding, activation agent, and target adsorbate used, playing a significant role in the activation stage (Kalderis et al. 2008). The most important thing is to determine the correct temperature and time, producing the significant specific surface areas, adequate pore formation, and surface functional groups without reducing the mechanical stability of the AC. The activation can be done through physical, chemical, or physiochemical routes. Table 3 summarizes selected studies on the activation of biomass through these synthetic routes.

**Table 3:** Activation of different biomass precursors through under different conditions

Biomass	Activation/condition	AC property			Ref
		*S*_BET_ (m^2^/g)	*V*_tot_ (cm^3^/g)	APD (nm)	
Physical					
Avocado kernel seeds	- CO_2_ flow, 1000 °C for 2 h	299.9 0.67	0.172		Shin et al. (2021)
Sugarcane	- CO_2_ flow, 900 °C for 4 h	906.1	0.174	1.505	da Cunha Gonçalves et al. (2016)
Alder wood	- Superheated steam, 850 °C for 90 min	1338 2.63	0.88		Rizhikovs et al. (2012)
Raw pine s	- CO_2_/N_2_ (1:1), heated up to 800 °C for 2 h	281 0.74	0.24		Plaza et al. (2015)
Chemical					
Hydrochar Microalgae	KOH; *R*_I_ = 1.5; 675 °C for 2 h under N_2_ flow	2099 5.9	1.2		Masoumi and Dalai (2020)
Date press cake	NaOH; *R*_I_ = 2꞉1; 650 °C for 2 h under N_2_ flow	2025.9 1.839	0.932		Norouzi et al. (2018)
Peanut shell	H_3_PO_4_; *R*_I_ = 1.5; 650 °C for 2 h under N_2_ flow	965.6	–	–	Garg et al. (2019)
Sunflower seed hull	K_2_CO_3_; *R*_I_ = 1꞉1.15; microwave power, 600 W, irradiation time, 8 min	1411.5	0.836	2.361	Foo and Hameed (2011)
Physicochemical					
Raw pecan shell	- KCl, 700 °C at 10 °C/min under N_2_ flow - Chemically activated precursor heated to 700 °C at 10 °C/min under CO_2_ for 2 h	808 2.672	0.546		Aguayo-Villarreal et al. (2017)
Raw coffee residue	- KOH; *R*_I_ = 3꞉1; 105 °C for 24 h - Chemically activated precursor heated to 700 °C at 10 °C/min under N_2_ and CO_2_ flow	1053	1.23	–	Giraldo and Moreno-Piraján (2012)
Raw palm shell	- H_2_PO_3_; *R*_I_ = 9.42; heated at 405 °C for 2 h at 5 °C/min under N_2_ flow - Chemically activated precursor heated at *T* = 855 °C under CO_2_ flow for 135 min	642 1.766	0.28		Arami-Niya et al. (2012)
Palm empty fruit bunch	- KOH; *R*_I_ = 1꞉1; for 2 h at 85 °C - Chemically activated precursor heated to 800 °C at 10 °C/min under CO_2_	720.00 1.889	0.34		Nasri et al. (2015)

*APD* average pore diameter

#### Physical activation

Physical activation is a two-step process, beginning with carbonization, followed by an activation at high temperatures in the presence of an oxidation agents such as steam, CO_2_, air, or a combination of them (Yin et al. 2014; Balahmar et al. 2017). Physical activation is primarily affected by the type of carbon precursor, particle size, gas flow, heating rate, activation time, and temperature and activating agent. It impacts on the produced carbons’ surface properties and porosity. According to Aworn et al. high-density materials, like shells and seeds, are the best activated using steam, whereas fibrous and low-density materials, like straws and husks, are better suited for CO_2_ activation. However, physical activation has disadvantages, including a high activation temperature, a prolonged processing time, a limited carbon yield, and a limited specific surface area (Aworn et al. 2008).

#### Chemical activation

In chemical activation techniques, biomass is impregnated with activating chemical agents. These reagents combine decomposition, dehydration, and complexation with organic carbon molecules of precursor materials. Subsequently, the chemically treated biomass is heated in an inert environment to produce activated carbons with a well-developed porous structure and a large surface area (Danish and Ahmad 2018). Moreover, the introduction of pores or surface chemical groups depends on the activating chemicals during activation. Alkaline, acidic, neutral, and self-activating chemical regulators have all been employed. Alkaline activating agents are divided into three types, namely (i) strong activators, including KOH and NaOH; (ii) mid activators, such as K_2_CO_3_ and Na_2_CO_3_; and (iii) weak alkaline activating agents, such as K_2_SiO_3_, Na_2_SiO_3_, K_2_B_4_O_7_, C_4_H_6_K_2_O_7_, CH_3_COOK, and C_6_H_5_K_3_O_7_ (Arslanoğlu 2019). According to various previous studies, KOH has been the most useful alkaline agent to create super-activated carbons (Gao et al. 2020; Jawad et al. 2021). Most of this agent will be transformed into K_2_CO_3_ and K_2_O during the activation process: carbon atoms reduce these two byproducts to form the metallic vapor potassium, responsible for pore developments (Arslanoğlu 2019).

- Acidic activating agents include H_3_PO_4_, H_4_P_2_O_7_, HPO_3_, H_6_P_4_O_13_, C_6_H_18_O_24_P_6_, and H_2_SO_4_. One of the successful acidic activating agents is H_3_PO_4_ because of its positive effects on textural and surface chemistry. At heating treatment above 217 °C, polyphosphoric acid is formed by condensing two or more H_3_PO_4_ molecules with the elimination of H_2_O. This formed agent exhibits strong corrosivity, which boosts volatile components’ oxidation and carbonization, creating more pores or transforming micropores into mesopores (Liu et al. 2012; Gao et al. 2020).
- Neutral activating agents include FeCl_3_, CaCl_2_, MgCl_2_, NH_4_Cl, K_2_SO_4_, KMnO_4_, KHC_4_H_4_O_6_ NaNH_2_, NaH_2_PO_4_ and KH_2_PO_4_, etc. Yuan et al. reported that the development of pores using neutral activating agents is directly related to the reaction between the positive ions (e.g., Fe^3+^, Mn^7+^, Mg^2+^, Na^+^) at their oxidation state with carbon atoms via reduction reaction (Gao et al. 2020).
- Self-activating agents include potassium gluconate, ferric ammonium citrate, potassium gluconate, sodium gluconate, sodium alginate, sodium tartrate, zinc citrate, potassium citrate, trisodium citrate, and a combination of citrate salts. Fuertes et al. reported that using sodium alginate, potassium gluconate, sodium citrate, and sodium gluconate as the carbon source and self-activating agent led to produce highly porous activated carbons with surface area ranging from 660 to 1040 m^2^/g (Fuertes et al. 2014). In another study, Dai et al. produced a porous carbon sheet using potassium acetate as both precursor and self-activating agent (Dai et al. 2018). The production of activated carbon using self-activating agents would solve the problems observed in chemical activation routes, such as the equipment corrosion and generating unwanted secondary pollution.

#### Physicochemical activation

The physicochemical activation integrates both physical and chemical processes, implying that carbon precursors are first chemically impregnated with activating agents before being physically activated in the presence of oxidizing gas. The advantages of physicochemical activation include its superior surface modification and controlled textural character. The complexity and high energy demands of physicochemical activation would limit its wide application.

### Synthesis of advanced carbon materials from biomass

Numerous investigations on activated carbon materials with various zero-dimensional (0D) fullerene, one-dimensional (1D) carbon (CNTs and carbon fibers), two-dimensional (2D) carbon (graphene, graphene oxide, and reduced graphene oxide), and three-dimensional (3D) graphene skeleton have been conducted (Gong et al. 2016, Ovais et al. 2022). Earlier studies on the synthesis of advanced carbon materials applied different methods, such as laser ablation system, chemical vapor deposition, and arc-discharge technique with the employment of catalysts like Co, Ni, and Fe and also by utilizing non-renewable carbon sources such as benzene and other external precursor carbon gases like ethylene and methane (CH_4_) that are generated from petroleum and coal products (C_2_H_4_). However, these procedures have certain restrictions as these nanostructures are made using non-renewable precursors, resulting in toxic waste and adverse environmental effects. Consequently, technologies must be improved for a non-toxic, more viable, and low-cost production of carbon nanostructures obtained from an eco-friendly environment. Biomass’s high carbon content, sustainability, and availability have made it a promising feedstock for producing value-added chemicals and sustainable carbon products.

**Fig. 4:**
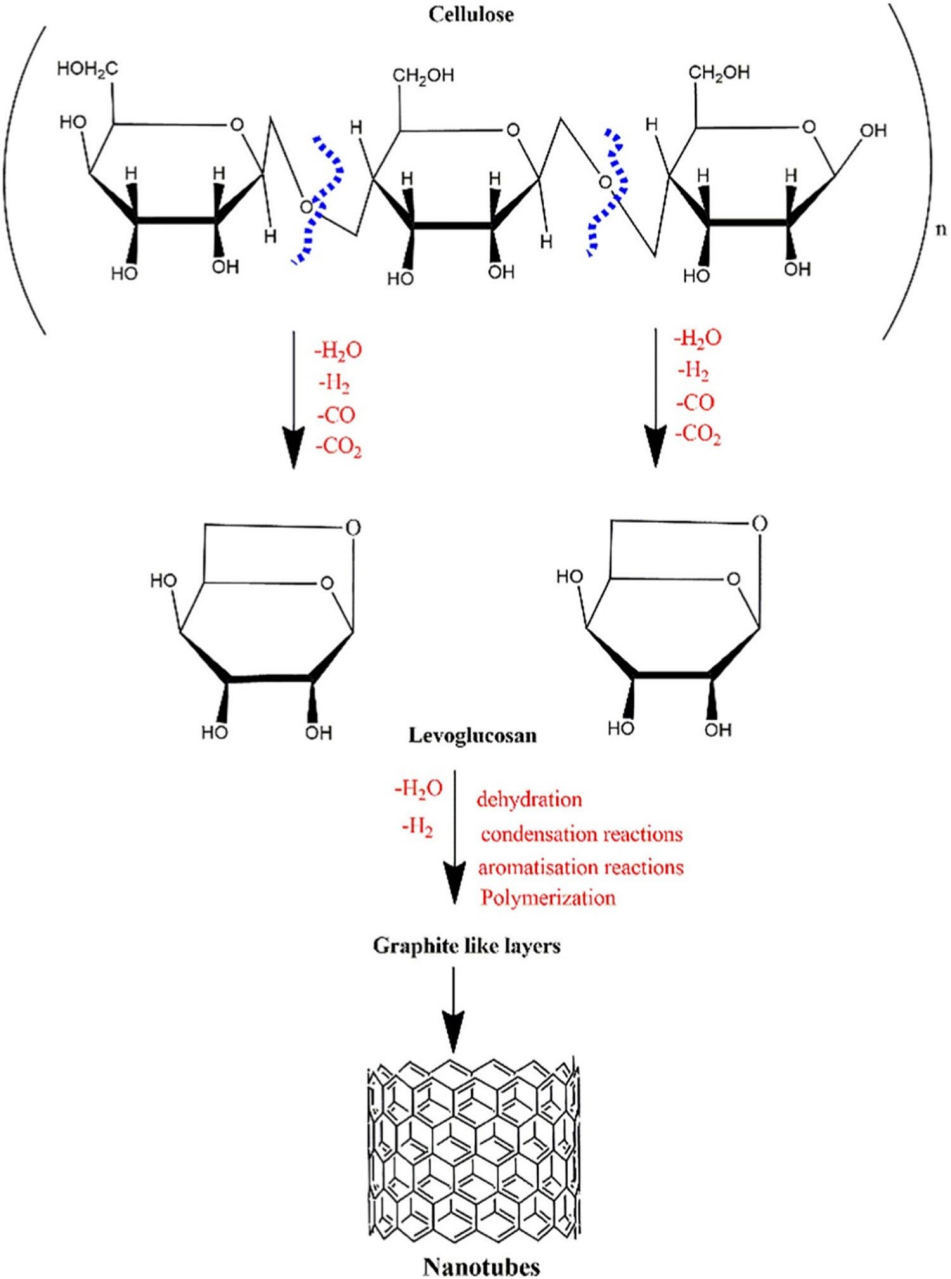
Reaction pathways during the decomposition of cellulose; copyright 2023 Elsevier (Omoriyekomwan et al. 2021)

A lot of research studies have demonstrated that lignocellulosic biomass is an appropriate bio-feed for producing nano-advanced carbon materials such as CNFs and CNTs. It comprises a substantial quantity of cellulose, which several studies have determined to be the precursor for developing CNTs (Sevilla and Fuertes 2010; Omoriyekomwan et al. 2019; Teo and Wahab 2020). Joy et al. proposed a mechanism for the transformation of biomass to CNTs via microwave-assisted pyrolysis; D-glucopyranose, a monosaccharide produced by the breakdown of cellulose, served as a carbon source for the production of CNTs (Omoriyekomwan et al. 2019). Anhydrides, oligosaccharides, and levoglucosan are produced when the glycosidic connections in D-glucose are broken (Zhang et al. 2011), and sugars are further broken down, fissured, and rearranged (Cho et al. 2010). The cleavage of the C–O bonds in levoglucosan is proceeded by aromatization to create layers that resemble graphite, as shown in Fig. 4 (Collard and Blin 2014; Muley et al. 2016). Table 4 shows selected studies on the production of advanced carbon materials from different biomass wastes.

**Table 4:** Selected studies on the production of advanced carbon materials from different biomass substrates

Biomass	Synthesis method/condition	Product	Ref
Coconut coir dust	Hydrothermal carbonization pretreatment and conventional pyrolysis 1500 °C	Carbon nanosheets	Barin et al. (2014)
Spruce bark	Hydrothermal treatment and conventional pyrolysis KOH activation 800–1000 °C	Graphene nanosheets	Wang et al. (2018)
Rice straw	Conventional CVD ferrocene and nickel nitrate catalyst 800 °C	CNTs	Fathy (2017)
Perilla frutescens	Conventional pyrolysis 600–800 °C	Carbon nanorods	Liu et al. (2017)
Camphor leaves	Conventional pyrolysis 1200 °C	Graphene sheets	Acharya et al. (2015)
Sawdust char	Conventional CVD FeCl3 catalyst 600–800 °C	Nanofibers	Liu et al. (2014)

Several studies report that the inorganic elements co-existing in biomass can catalyze the production of CNT or MWCNT products. By illustrating a single-step synthesis strategy to generate MWCNT, Ramya Araga et al. have successfully established the importance of minerals contained in biomass as catalysts (Araga and Sharma 2017). This method for creating CNTs on the surface of coconut shell-derived carbon does not require extra impregnation of metal nanoparticles. Zhu et al. reported that the use of Mg_2_SiO_4_ is the reason behind the nucleation and growth of CNTs (Zhu et al. 2012). The same results were shown by Shi et al. demonstrating the effect of SiC in the nucleation of MWCNT without the need for any additional catalysts (Shi et al. 2014).

## Adsorption of pollutants in water phase

### Removal of organic pollutants from water

**Fig. 5:**
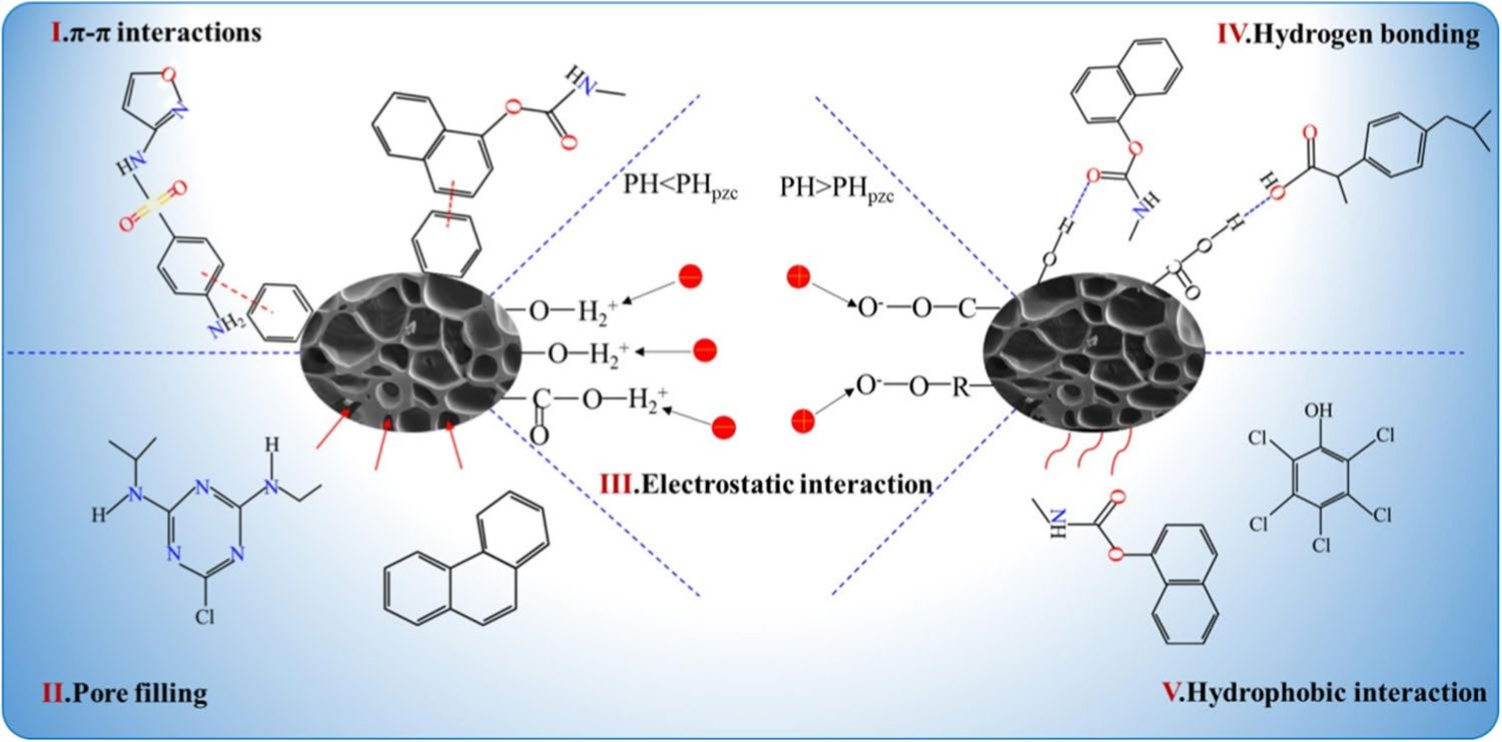
Common mechanisms of organic pollutants adsorption on the surface of carbon materials, reproduced with permission from Qiu et al. (2022); copyright 2023 Elsevier

Rapid industrial and agricultural activities have resulted in the discharge of organic pollutants, such as dyes, persistent organic pollutants (POPs), polycyclic aromatic hydrocarbons (PAHs), pharmaceuticals, pesticides, and industrial chemicals into the aquatic environment. Indeed, the adsorption process is a general approach to physically remove these compounds from water and wastewater (Galloni et al. 2022a, b; Galloni et al. 2022a, b). Activated carbon is recognized as one the most widely applied adsorbent for the adsorption of organic pollutants during large-scale applications. Numerous researches on the conversion of biomass-based wastes into carbon materials have been published over the past few decades, along with various surface modification techniques to improve selectivity and adsorption yield (Nor et al. 2013, Arun et al. 2021; Jjagwe et al. 2021; Shukla et al. 2021). In terms of mechanistic pathways, several adsorptive routes have been reported that in general depend on the surface chemistry of the adsorbent (functionalization, wettability, porosity, surface area, and so on), the nature of the organic pollutants and also the working conditions, i.e., pH of the solution. Overall, five adsorption pathways could be classified as shown in Fig. 5.

- Physical pore filling route: the accumulation of organic molecules inside the pores of biomass-based absorbent is the standard way to remove organic pollutants from water. In this regard, the produced activated carbon must have a high porosity. Additionally, the relationship between the size of the pores and of the organic pollutant is crucial.
- Electrostatic interaction–based adsorption is based on the attraction or repulsion between the ionic surface of the adsorbent and the ionizable organic molecules. The surface charge and organic pollutants are important components in this scenario. The formation of repulsion forces is caused by the same sort of charge, while opposing charges enhances the fixation of organic pollutants. In this adsorption process, the pH of the solution, which may directly influence the ionic state of the adsorbent surface and organic molecule, is important because acidic or basic modification of the adsorbent surface greatly increases the electrostatic attraction.
- Hydrogen bonding–based adsorption: this adsorption process is based on the intermolecular or intramolecular interactions between hydrogen and other atoms, such as nitrogen, oxygen, chlorine, fluorine, and bromine. Similar to electrostatic attraction–based adsorption, carbon materials with abundant oxygen functional groups promote the interaction with hydrogen, resulting to adsorption by hydrogen bonding. Additionally, this interaction makes it simple to fix highly polar organic contaminants on the adsorbent surface.
- Hydrophobic-based adsorption: this mechanism correlates both electrostatic attraction and distribution. The fixation of hydrophobic organic pollutants occurs mostly on the surface of hydrophobic adsorbents. Therefore, the modification of biomass-based carbon materials with hydrophobic agents is commonly used.
- π–π binding–based adsorption: aromatic organic pollutants with C–C conjugation can form π–π interactions with aromatic structures present in some carbon materials. Oxygen, nitrogen, or amine-based groups can boost π–π interactions.

Based on the above discussion, the adsorption of organic pollutants is dependent on a number of variables, including the surface chemistry and textural porosity on the one hand, and the properties of the organic contaminants and working circumstances on the other. It is important to note that the adsorption of organic pollutants cannot be accomplished by a single process, but rather through a combination of the several mechanisms mentioned above, with one or two mechanisms predominating.

Table 5 lists the adsorption capacities of carbon materials prepared from different biomass substrates to remove various organic pollutants.

**Table 5:** Adsorption capacities of selected carbon materials prepared from different biomass substrates towards organic pollutants removal from water

Biomass	Pollutant	Adsorption capacity	Ref
Straw	Ethyl acetate	240.4 mg/g	Li et al. (2021)
Wheat	Triclosan	529.1 mg/g	Guérin et al. (2020)
Sunflower	Methylene blue	379.0 mg/g	Hubetska et al. (2021)
Wheat straw	Ciprofloxacin	909.09 mg/g	Wang et al. (2020a, b, c, d, e, f)
Palm kernel	Atenolol Acebutolol Carbamazepine	0.69 mmol/g 0.67 mmol/g 0.72 mmol/g	To et al. (2017)
Spent coffee waste	Methylene blue, tetracycline	812 mg/g 462 mg/g	Ahsan et al. (2018)
Physalis peruviana	Ketoprofen 2,4-dichlorophenoxyacetic	172 mg/g 209 mg/g	Dhaouadi et al. (2022)
Rice husk	Phenol	8.086 mg/g	Meng et al. (2021)
Pomelo peel	Oils and organic solvents	23–48 g/g	Liu et al. (2022)

**Fig. 6:**
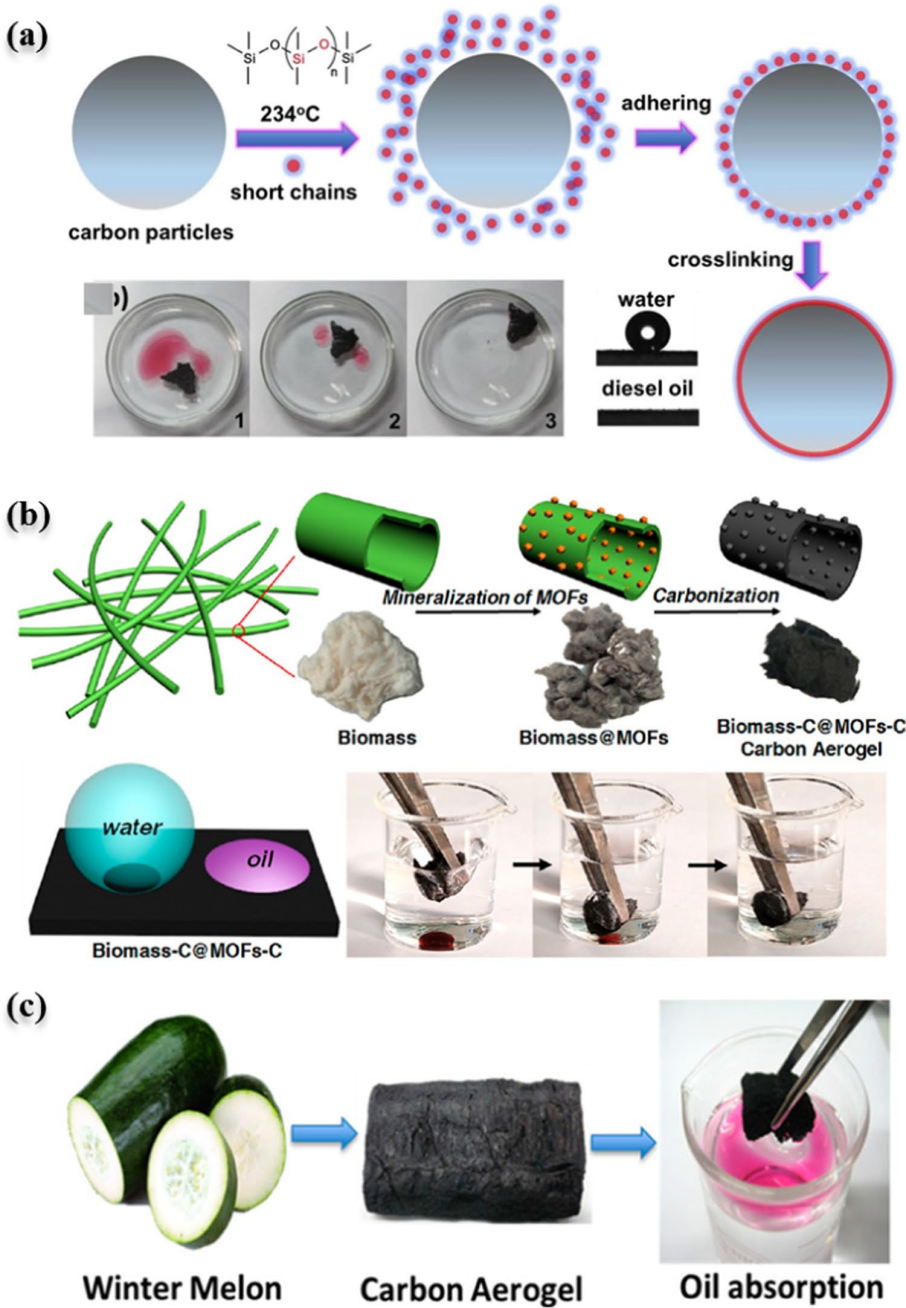
**a** Modification of sunflower-based activated carbon with PDMS for enhanced hydrophobicity towards the selective adsorption of organic and oily pollutants over water molecules, diesel oil separation from (dyed with red oil O)/water mixture (left 1–3), reproduced with permission from Sun et al. (2019). **b** Steps of the synthesis of biomass-C@MIL-53-C for the adsorption of oily pollutants in water, reproduced with permission from Zhao et al. (2020). **c** Valorization of winter melon as carbon aerogel for the oil adsorption in water, reproduced with permission from Li et al. (2014)

Surface modification to aid the fixation of organic pollutants has frequently been reported. Adsorbent surface hydrophobicity alteration is one method for solving the co-adsorption of water molecules, which typically lessens the fixation of organic contaminants. Sun et al. (2019) modified sunflower biomass–based activated carbon with polydimethylsiloxane (PDMS) to increase the hydrophobicity (Fig. 6a). Study also suggests that the incorporation of porous materials, such as MOFs, could provide a hierarchically structured surface to the biomass-based activated carbon to enhance the adsorption capacity and the selectivity towards the organic pollutants. For instance, Zhao et al. (2020) modified raw kapok fibers biomass by MIL-53 before carbonization to obtain hierarchically structured activated carbon (Fig. 6b) for the adsorption of oils and organic solvents in water. MOF-modified biomass displayed superhydrophobic features compared to raw biomass based on the contact angle results. Similarly, the novel biomass-C@MIL-53-C exhibited superior adsorption capacity that is reportedly 119 times higher than bare biomass-C towards the removal of oils/organic solvents, including n-hexane, chloroform, and so on. Carbon aerogel prepared from winter melon was reported to have high adsorption of oil in the water system (Fig. 6c) (Li et al. 2014). Surface modification of biomass by kaolinite clay was reported by Olu-Owolabi et al. (2017) for the adsorption of 2,4,6-trichlorophenol in water. It was noticed that the surface area decreased by 57% compared to bar kaolinite; however, the ion-exchange capacity increased by fourfold. The adsorption capacity increased by up to 250% compared to bare kaolinite due to the presence of functional groups, proving more electrostatic interactions with the pollutant.

Magnetic reusable activated carbon made from biomass has drawn a lot of interest as absorbent recovery is one of the challenges associated with suspension-based adsorption treatment. The application of magnetic materials facilitates the recovery of suspension by applying an external magnetic field. Therefore, recycling the adsorption suspension would be easy (Mehta et al. 2015; Tamjidi et al. 2019). A number of methods, including co-precipitation, hydrothermal, thermal decomposition, sol–gel, chemical reduction, pyrolysis, polyol, and microwave-assisted pyrolysis, have been described to combine magnetic iron with biomass-based carbon materials as reviewed by Noor et al. (2017). Sirajudheen et al. reported the synthesis of MnFe_2_O_4_ *Moringa oleifera* seed–based carbon material for the adsorption of organic dyes (Sirajudheen et al. 2021). For this, firstly *Moringa oleifera* seeds were carbonized, and then, the produced activated carbon was coated by MnFe_2_O_4_ through a simple co-precipitation route. The authors report a significant adsorption capacity that was obtained by magnetic adsorbent as compared to barely activated carbon adsorbent and MnFe_2_O_4_.

### Removal of heavy metals from water

**Fig. 7:**
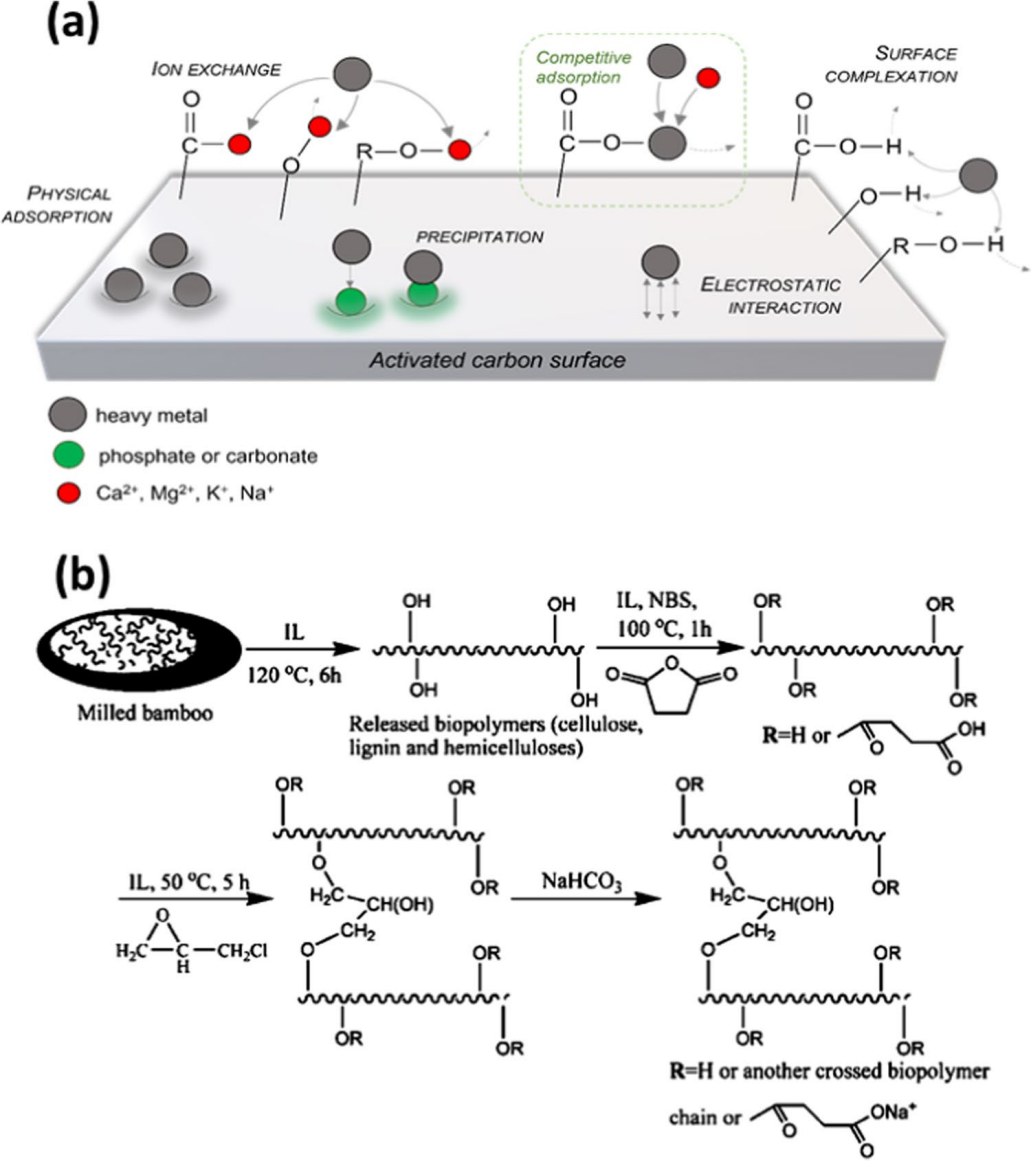
**a** Adsorption mechanisms of carbon-based materials for heavy metal removal. **b** Dissolution of biomass and reconstruction in ionic liquid to fabrication of effective bioadsorbent for heavy metal adsorption, reproduced with permission from Zhong et al. (2012)

Over the years, biomass-based activated carbons have been widely studied as sorbents for the selective removal of several heavy metal ions. In particular, different modification processes (i.e., magnetization, oxidation, functional group grafting, and construction of composite inorganic materials) are recognized to be effective strategies for enhancing the adsorbent capacity of activated carbons (Abdolali et al. 2015; Yang et al. 2019; Šoštarić et al. 2020; Shukla et al. 2021). However, the adsorption capacity of activated carbons is driven by different adsorption mechanisms (Fig. 7a), categorized into five classes namely, (i) physical adsorption, (ii) ion exchange, (iii) electrostatic interaction, (iv) surface complexation, and (v) precipitation.

Physical adsorption, which is independent of the holding power of the chemical bonds, leads the distribution and dispersion of heavy metal species into the pores of the adsorbent material. In fact, the pore size distribution and surface area of the adsorbent play a major role in this adsorption. In particular, the higher the surface area and the mesopores, the quicker the pollutant dispersion onto the adsorbent surface with consequent enhancement of the whole adsorption phenomenon (Ma and Wang 2020; Hoang et al. 2022). Ion exchange is a process during which the exchange of ions between the functional groups containing oxygen species (e.g., –OH, –COOH) and divalent metal cations (M^2+^) happens. In particular, it is known as the primary adsorption phenomenon between heavy metal species and activated carbons (Yang et al. 2019; Hoang et al. 2022). Electrostatic interaction has been recently classified as another adsorption mechanism, as reported by Yang et al. (2019). In particular, it occurs when the adsorbent’s surface is negatively or positively charged. In this way, an electrostatic attraction force is created, favoring the oppositely charged metal ions towards the adsorbent material. It is crucial to note that the surface properties of carbonaceous materials affect the occurrence of various charges; hence, the pH value and the isoelectric point (pH_pzc_) are paramount parameters to evaluate (Wang et al. 2014). Surface complexation of different functional groups consists in the interaction between the functional groups (e.g., –OH, O^2−^, –CO–NH–, and –COOH) of the adsorbent and the heavy metal ions, generally M^2+^ or complexes (i.e., Pb(OH)^+^, Cd(OH)^+^) (Li et al. 2017). In this way, multi-atom structures are formed as a consequence of this phenomenon (Li et al. 2017). Eventually, precipitation or coprecipitation results in formation of solidified participles as a consequence of interactions between heavy metal ions and the adsorbent surface. The process typically occurs in an aqueous solution containing high levels of heavy metal concentrations (Liu et al. 2020).

The reduction process is crucial for the extraction of heavy metals when there are high-valence heavy metal ions present. Heavy metal ions are, in fact, initially reduced to low-valence states before being eliminated by surface complexation or ion exchange pathways (Li et al. 2017). The above-described mechanisms resume all the possibilities in which carbon-based materials can remove heavy metal ions. The physicochemical characteristics of the adsorbent, the properties of heavy metal ions, and the experimental settings in which the adsorption phenomenon occurs all have a significant role in how effective the adsorption capacity is in this context (e.g., pH, initial concentration of heavy metal ions, presence of coexisting ions, temperature).

To improve the adsorption ability, most of the studies report surface modification by chemical agents to functionalize the surface for enhanced fixation of heavy metal ions through the mechanistic pathways reported above. Li et al. (2022a, b) modified *Medulla Tetrapanacis* biomass with nitrogen (from urea) and NaHCO_3_ during its pyrolysis at 700 °C. N-rich biochar showed excellent sorption capacities of 468.5 mg/g and 1466.5 mg/g for Cu^2+^ and Pb^2+^, respectively. Heavy metal fixation occurs through mechanistic mechanisms such physical adsorption, complexation, and precipitation. Citric acid is applied to the surface of biomass-based adsorbents to enrich the surface with oxygenated functional groups, which enhances the fixation of heavy metal cations (Monroy-Figueroa et al. 2015). For the fixation of heavy metal cations, adsorbents with negatively surfaces can promote the fixation ability. Ali Khan et al. (2017) developed a dodecyl sulfate chain-anchored mercerized lignocellulosic biomass, which enhances the electrostatic interactions with heavy metal cations. The authors report a very rapid fixation of Pb^2+^, Cd^2+^, and Zn^2+^ on the negatively charged biomass. Lignocellulosic biomass can be broken down and rebuilt to improve its sorption capacity. For example, Zhong et al. have dissolved milled bamboo biomass in IL 1-ethyl-3-methylimidazolium acetate and then reconstructed it in ionic liquid (saturated sodium bicarbonate), to build cross polymers, as shown in Fig. 7b. The authors reported that the maximum adsorption capacities of Pb^2+^ and Cd^2+^ were 381.7 and 278.6 mg/g, wherein an ion exchange process was the adsorption mechanism on the surface of bio adsorbent.

## Adsorption of pollutants in air phase

Carbon materials are widely used to remove several air pollutants through adsorption (Raso et al. 2014; Ligotski et al. 2019; Wang et al. 2020a, b, c, d, e, f; Roegiers and Denys 2021; Maximoff et al. 2022). Recently, the valorization of biomass-based materials into activated carbon or biochar towards the adsorption or/and reduction of air pollutants, including VOCs, NO_x_, CO_2_, and so on, has been extensively studied. This section discussed biomass-based activated carbon materials for air treatment by stressing modification and activation routes.

### VOC adsorption

VOCs are primarily discharged from industries, such as organic chemicals, printing, and paint, with considerable adverse effects on the environment (Wang et al. 2013; Liu et al. 2019a, b). The adsorption of VOCs on activated carbons depends on different factors, including the surface area, pore size, and the surface functional groups from one side, and characteristics of volatile molecules such as polarity, weight, and hydrophobicity (Lucena et al. 2012; Huang et al. 2021; Rahbar-Shamskar et al. 2022). Biomass-based activated carbon materials were used as adsorbents to capture VOC materials (Ikram et al. 2022). The improved surface function enables greater chemical interactions, surface modification is a crucial role in enhancing VOC fixation. Shen et al. (2020) reported that the activation of corncobs-derived carbon adsorbent with KOH improves the selectivity of VOC adsorption (851.3 and 854.9 mg/g for benzene and toluene, respectively) three times better than the adsorption capacities using a commercial activated carbon. Li et al. (2011) studied the chemical modification of coconut shell–based activated carbon towards the fixation of hydrophobic VOC (o-xylene). It was reported that the acid modification, using sulfuric acid and phosphoric acid, decreased the adsorption capacity of o-xylene. In contrast, the modification by alkalis (ammonia and sodium hydroxide) boosts the adsorption of o-xylene.

The applied pressure during the adsorption of VOCs by activated carbon-based materials has a significant role depending on the pores size. Ma et al. (2021) developed biomass-based hierarchical porous carbon with highly surface area of 3936 m^2^/g via KOH-combined pyrolysis. The adsorption of VOCs (acetone and methanol) under high pressure, according to the authors, is controlled by the overall pore volume. The adsorption of VOCs is also controlled by surface oxygen, functional groups, and electrostatic interactions at low pressure. Another important consideration is the VOC molecule’s structure. It was reported by Li et al. (2022a, b) that the micropores and heteroatom influence the oxygenated VOCs adsorption behavior on biomass-based hierarchical porous carbon at low pressure. However, the adsorption of aromatic-based VOCs depends on different sizes at various applied pressures, as summerized in Table 6.

**Table 6:** Selected studies on the use of biomass-based adsorbents for VOC removal

Biomass	Targeted pollutant	Adsorption capacities	Main conclusions	Ref
Pine sawdust AC	Toluene Acetone	681.8 mg/g 570 mg/g	Microwave KOH activation resulted in highly adsorptive AC at room temperature with relative pressures between 0.01 and 0.9	Mao et al. (2016)
AC-corn-cob	Toluene	388.8 mg/g	AC-corn-cob was prepared under different conditions. The porosity and functional groups exhibit a direct effect on toluene adsorption	Zhu et al. (2018)
Ac-corn-cob	Toluene	414.6 mg/g	The chemical activation using ZnCl_2_ was the reason behind the enhanced adsorption performance	Zhu et al. (2018)
Waste shaddock peel	Benzene Toluene	16.58 mmol/g 15.50 mmol/g	The adsorption capacity was higher than those obtained using zeolite and commercial AC. In addition, an enhanced thermal capacity was recorded	Yang et al. (2022)
Straw-based AC	Ethyl acetate Toluene	240.4 mg/g 321.9 mg/g	The straw-based activated carbon showed excellent adsorption behavior and regeneration	Li et al. (2021)
Coconut shell AC	o-xylene	305.70 mg/g	Alkaline modification (ammonia or sodium hydroxide) boosts the adsorption of hydrophobic VOC (o-xylene), while the modification by acid modification (sulfuric or phosphoric acid) reduces the adsorption	Li et al. (2011)
Coconut shell AC	Benzene Methanol *n*-hexane Cyclohexane	1846 mg/g 1777 mg/g 1510 mg/g 1766 mg/g	The excellent adsorption behavior is due to the textural properties of obtained AC	Zhang et al. (2020a, b)
Hierarchical porous carbon	Acetone Methanol	26.1 mmol/g 46.9 mmol/g	The total pore determines VOC adsorption at high pressure, and surface oxygen functional groups determine VOC adsorption at low pressure	Ma et al. (2021)
Hierarchical porous carbon	Acetone	22.4 mmol/g	Oxygenated VOC adsorption depends on the micropores and heteroatom at low pressure Aromatic VOC adsorption depends on different sizes at various applied pressures	Li et al. (2022a, b)

### NO_x_ adsorption and reduction

Nitrogen oxides (NO_x_) seriously affect human health and the environment. For decades, NO_x_ pollution has been considered one of the environmental issues that requires an urgent investigation to develop approaches for their removal or/and reduction (Sun et al. 2016; Damma et al. 2019; Zhang et al. 2020a, b). Removal of NO_x_ via adsorption using biomass-based activated carbon materials has been a hot topic recently (Reza et al. 2020). Wang et al. (2010) investigated the effect of biomass type (wheat straw, char of wheat straw, cotton stalk, and rice husk) on the reduction of NO_x_ during the re-burning of these wastes. Consequently, the heterogeneous reduction of NO reached approximately 59% total NO_x_. Their results also showed the different biomass behaved differently toward reducing NO_x_. For instance, out of the different biomass fractions, NO reduction followed the order cotton straw > wheat straw > rice husk. Hence, the appropriate selection of biomass is essential for effectively removing NO_x_. Other studies have shown that using biomass or coal as re-burning fuels could heterogeneously reduce NO using pyrolytically produced char at high temperatures (Zhong et al. 2002; Zhang et al. 2007; Guo et al. 2019). Shu et al. (2018) investigated a series of herbaceous and lignocellulose biomass as activated carbon support for the subsequent reduction of NO_x_ to N_2_. The authors examined the effect of carbonization temperature, biomass type, and catalyst composition on NO_x_ reduction. They reported that NO_x_ adsorption onto carbon was higher at a temperature lower than 250 ºC in all materials. Although enhanced reaction adsorption at low temperatures is achievable, high flue gas temperatures and low levels of acidic gas can present a major bottleneck, resulting in low adsorption capacities and poor selectivity (Abdulrasheed et al. 2018). Modifying the surface of activated carbon is one way to help increase the formation of immobilized functional groups that improves NOx removal from air. For example, the selective reduction of NO_x_ at higher temperatures could also be achieved via the addition of an alkali, such as KOH (Sumathi et al. 2010), transition, and alkaline-earth metals such as potassium (García-García et al. 1997; Illán-Gómez et al. 1998), calcium (Kordylewski et al. 2005), and nickel (Illán-Gómez et al. 1999). For instance, by impregnating a coconut shell-activated carbon with KOH, Sumathi et al. (2010) achieved a simultaneous reduction of both NO_x_ and SO_2_. Furthermore, the AC could access the pore structure after adding potassium, significantly enhancing the efficiency of NO_x_ and SO_2_ reductions due to the high reactivity on its surface.

The promotional effect of oxygen on the adsorption of NO_x_ when using activated carbon as a catalyst has been studied previously. Zhang et al. (2008) examined NO adsorption by activated carbon in the presence or absence of oxygen in a reaction having 500 mg/L NO and a temperature below 100 °C. Interestingly, a negligible NO reduction was recorded in the system without oxygen, but about 10% reduction was obtained with oxygen. The role of activated carbon is to first convert NO to NO_2_ that could readily be adsorbed. The catalytically oxidizing NO to NO_2_ could be a practical way of removing NO_x_ from flue gas since NO_2_ is readily removed by water. Loading an activated carbon with silver nanoparticles was a successful route to enhance the adsorption of NO_2_, promoting its conversion to NO and the subsequent conversion to N_2_ (Seredych et al. 2010). Additional tests using fed dry air containing 0.1% NO_2_ at room temperature showed that 0.40 mmol/g of NO_2_ was adsorbed.

### CO_2_ adsorption

Over the past few decades, with the continuous rise in CO_2_ concentration, a common greenhouse gas has been a source of serious environmental threat by serving as a heat nesting site in the atmosphere leading to global warming. A common method for fixing CO_2_ is adsorption. The yield of adsorption depends on its thermodynamic. It could be fixed on the surface through physisorption or chemisorption (Creamer et al. 2014; Lee and Park 2015). In physisorption, CO_2_ is fixed through weak van der Waal forces, whereas chemisorption occurs via stronger chemical bonds, which lead to higher adsorption capacity, but the regeneration is more difficult. Biomass-based activated carbon materials have been widely used for CO_2_ capture (Abuelnoor et al. 2021). Similar to VOCs and NO_x_, CO_2_ adsorption depends on the materials’ textural characteristics and functional groups and the working conditions, such as the pressure and temperature. To improve the selectivity of CO_2_ adsorption on activated carbon, studies have revolved around the synthesis and functionalization of activated carbon from different biomass materials. Manyà et al. (2018) prepared biochar from vine shoots through CO_2_-physical and KOH-chemical routes. The authors reported that the chemically activated biochar exhibits maximum adsorption of CO_2_ at 25 °C at an absolute pressure of 15 kPa, whereas the physical activation with CO_2_ shows maximum adsorption at 75 °C. In addition, these biochar adsorbents showed enhanced selectivity towards CO_2_ over N_2_. Another study, which compares the physical and chemical activation of biomass (spent coffee grounds) towards CO_2_ adsorption, was reported by Plaza et al. (2012). It was found that the KOH chemical activation led to the best CO_2_ uptake, whereas the CO_2_ physical activation leads to better CO_2_/N_2_ selectivity. Although surface area is a crucial component of CO_2_ adsorption, several studies have shown that increasing surface area is not the sole way to enhance CO_2_ fixation. Wang et al. (2012) prepared an activated carbon with an excellent surface area of 3404 m^2^/g via the valorization of celtuce leaves waste into activated carbon using air-drying, pyrolysis, and chemical activation by KOH. However, the fixation of CO_2_ was 6.04 and 4.36 mmol/g at 0 and 25 °C. This result is not very far from activated carbon-based materials with much lower surface area (Abuelnoor et al. 2021).

**Fig. 8:**
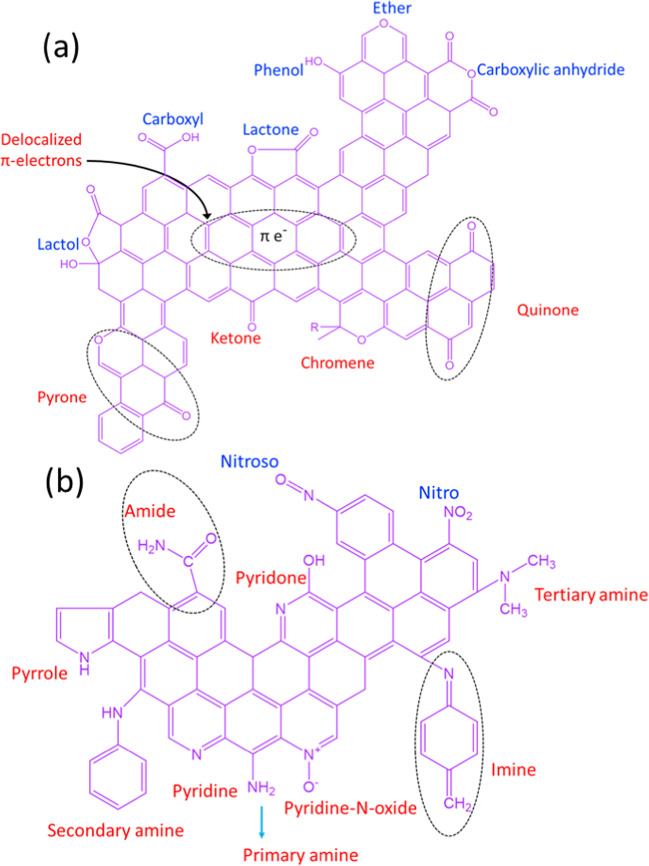
Common surface modification of activated carbon-based materials for boosting the fixation or air pollutants; **a** oxygen-based group modification, **b** nitrogen-based groups modification, reproduced with permission from Abdulrasheed et al. (2018)

**Fig. 9:**
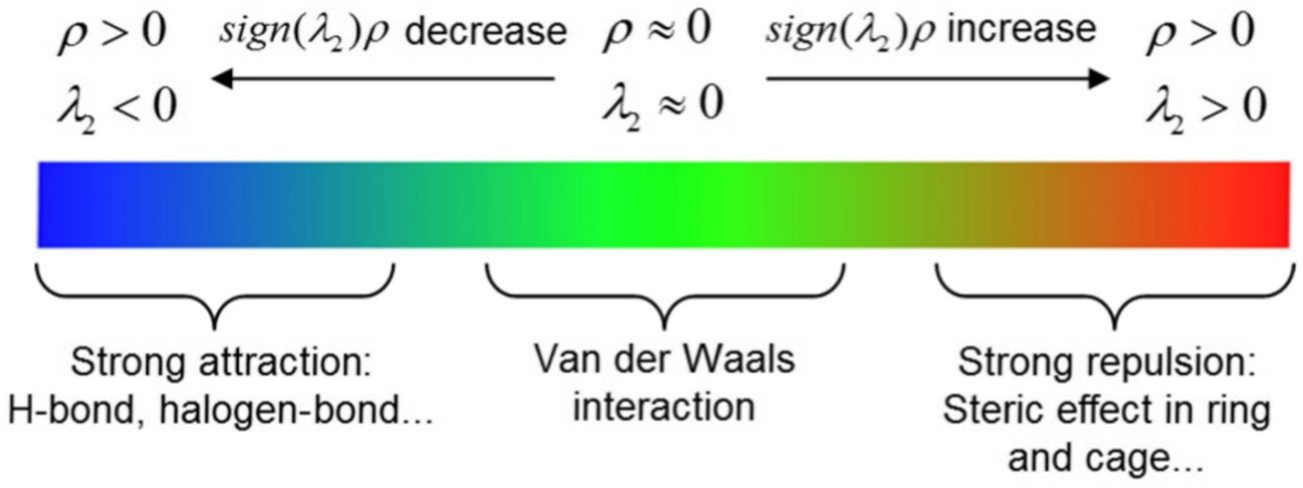
Types of interactions between air pollutants and activated carbon surface as a function of the symbol of the second largest eigenvalue (*λ*_2_) and *ρ* are the electron density, reproduced with permission from Wang et al. (2022)

The functionalization of activated carbon-based materials to enhance the fixation of CO_2_ is an integral approach (Arenillas et al. 2005; Pevida et al. 2008; Lee and Park 2015). In general, acidic, basic, or neutral heteroatoms are incorporated on the surface to modify their electronegativity and provide active sties for enhanced attraction of CO_2_ molecules (Shafeeyan et al. 2010). Figure 8 summarizes the most common surface modification of activated carbons by oxygen or nitrogen functional groups. For example, incorporating nitrogen into the surface of activated carbon could boost the fixation of CO_2_. Zhang et al. (2016a, b) concluded that the heat treatment of KOH-activated black locust with ammonia leads to the incorporation of nitrogen on the surface, which in turn boosts the fixation of CO_2_ as compared with bare KOH-activated black locust. Based on the surface functional groups and pollutants characteristics, different surface interactions or repulsion would take place as shown in Fig. 9.

### SO_2_ adsorption

**Fig. 10:**
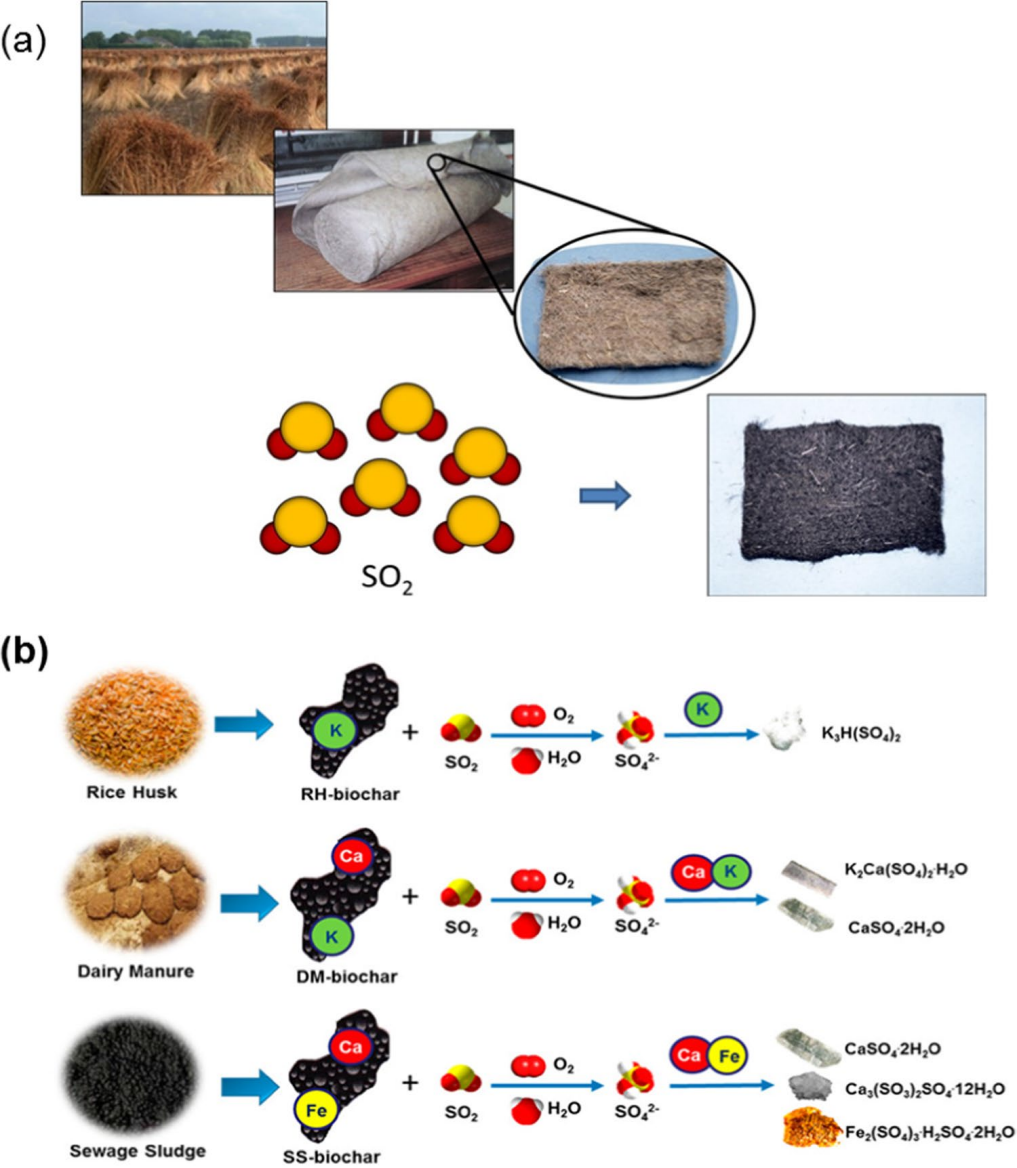
**a** Valorization of fibrous waste biomass (flax) into adsorptive membrane for SO_2_ fixation; reproduced from Illingworth et al. (2019). **b** Valorization of different biomass wastes into biochar for the adsorption and transformation of SO_2_, reproduced with permission from Xu et al. (2016)

The emission of SO_2_, a toxic gas, is a severe environmental issue nowadays. A lot of efforts have been made for the appropriate removal/reduction of SO_2_ in phase using different technologies, namely wet scrubbing (Zhao et al. 2021a, b), dry-based reduction technologies (Ng et al. 2022), and adsorption (Raymundo-Pinero et al. 2000; Kang et al. 2020; Liu et al. 2021). The valorization of biomass into activated carbon towards the reduction of SO_2_ removal was investigated by several studies. Illingworth et al. reported the synthesis of activated carbon from fibrous waste biomass (flax) for SO_2_ adsorption in a continuous-flow reactor (Fig. 10a) (Illingworth et al. 2019). The surface area increased with the carbonization temperature, which was found to be 126 and 1177 m^2^/g at 450 and 800 °C, respectively. In addition, a very microporous surface was obtained. The adsorption of SO_2_ increases with the surface of the materials, whereas the results showed that flax-based activated carbon has better adsorption than the commercial one. Wang et al. (2022) prepared an activated coke from valorizing mixed sewage sludge and waste biomass via carbonization and ammunition for SO_2_ adsorption. It was reported that the nitrogen-containing functional groups boost the chemical fixation of SO_2_. The presence of inorganic components in biomass-based materials can provide further reduction reactions on top of the adsorption. Zhang et al. (2020c) synthesized nitrogen-enriched biochar from different biomass materials and reported an adsorption capacity of up to 216.19 mg/g at 120 °C. Xu et al. (2016) prepared three types of biochar from different starting materials, including rice hush, dairy manure, and sewage sludge for the adsorption of SO_2_ from the air. The biochar-based materials showed adsorption capacities of about 8.87–15.9 mg/g. However, the increase of humidity content from 0 to 50% led to a threefold increase in adsorption capacities due to the formation of alkaline water functional groups to boost the fixation of SO_2_. Interestingly, it was claimed that biochar with higher mineral constituents led to better SO_2_ removal. As shown in Fig. 10b, mineral constituents can convert SO_2_ into SO_4_^2−^ that is converted into different mineral salts.

### Mercury adsorption

Biomass-based activated carbon materials were also used for the fixation of gaseous Hg^0^ (Skodras et al. 2007; Asasian et al. 2012; Egirani et al. 2021). Liu et al. reported the valorization of seaweed biomass into activated carbon for the adsorption of Hg^0^ in the air (Liu et al. 2019a, b). It was found that the fixation is controlled by mass transfer at 80 °C, whereas the increase in the temperature to 120 and 160 °C boosts the chemisorption. The high surface area and porosity aided the physisorption reaction. The investigation also proves that C–Cl and oxygen functional groups on the activated carbon surface promoted the chemisorption reaction. In another study [227], corn cob activated carbon was activated using ZnCl_2_. It was reported that both Zn and Cl, besides to enhance porosity and surface area, play an essential role in capturing of Hg^0^. Cl could boost the surface oxidation of Hg^0^ into its oxides, while Zn can increase tensile strength and water adsorption. Zhao et al. reported that the efficiency of Hg^0^ adsorption on the surface of biomass carbon and activated carbon depends on the pore structure and Br content (Zhao et al. 2021a, b). It was claimed that C–Br could oxidize the physically adsorbed Hg^0^ into HgBr_2_, then transform it to C–O–Hg, and finally desorb it as HgO. At temperatures ranging from 80 to 120 °C, the oxidation of the surface of biomass carbon is more pronounced. At the same time, further increase leads to an inhibited oxidation due to the breakdown of Van der Waals forces bonds. The bromination of biomass ash towards the enhanced usage of a chemical–mechanical approach was reported by Bisson et al. (2013). Arvelakis et al. (2010) studied the pretreatment role and biomass activation. The authors concluded that the untreated biomass exhibits better Hg^0^ adsorption than the lignite-activated carbon treated at 850 °C. It was claimed that the high calcination temperature leads to the decomposition of alkali groups, reducing the capture of Hg^0^. In addition, it was mentioned that the high surface area of the activated carbon does not seem to have a significant effect on the adsorption of Hg^0^.

**Fig. 11:**
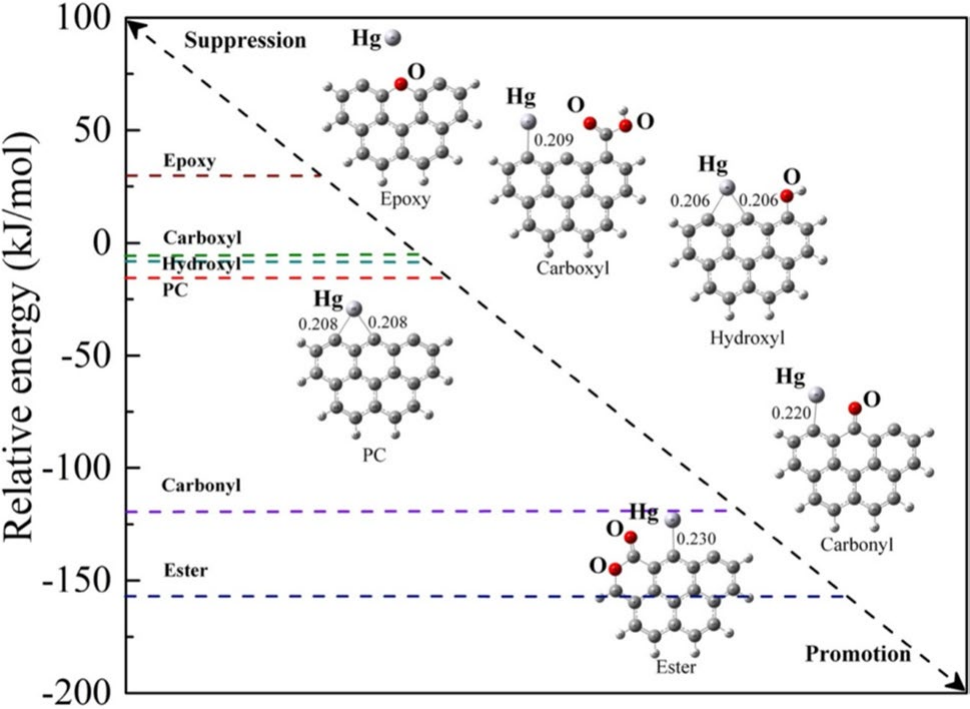
Adsorption energies of Hg.^0^ on different oxygen groups, reproduced with permission from Shen et al. (2018)

In terms of surface oxygen, both yield of atomic oxygen on the surface and nature of oxygen groups are determinants for the fixation of Hg^0^. Shen et al. studied oxygen groups’ role in the adsorption of Hg^0^ on the surface of biomass-based activated carbon (Shen et al. 2018). The treatment of the adsorbent surface via non-thermal plasma incorporated oxygen functional groups on the surface. The authors reported that carbonyl and ester groups boost the fixation of Hg^0^, while epoxy, carboxyl, and hydroxyl groups reduce the adsorption capacity (Fig. 11). The adsorption was improved after the non-thermal plasma treatment of the activated carbon due to the incorporation of ester and carbonyl groups. The promotion effect of the adsorption ability is due to the higher adsorptive energies of Hg^0^ on the carbonyl and ester groups than on the surface of activated carbon. At the same time, the suppression is because of the lower adsorption energies of Hg^0^ on the epoxy, carboxyl, and hydroxyl groups compared to that on the surface of activated carbon.

## Photocatalytic removal of organic pollutants from water

**Fig. 12:**
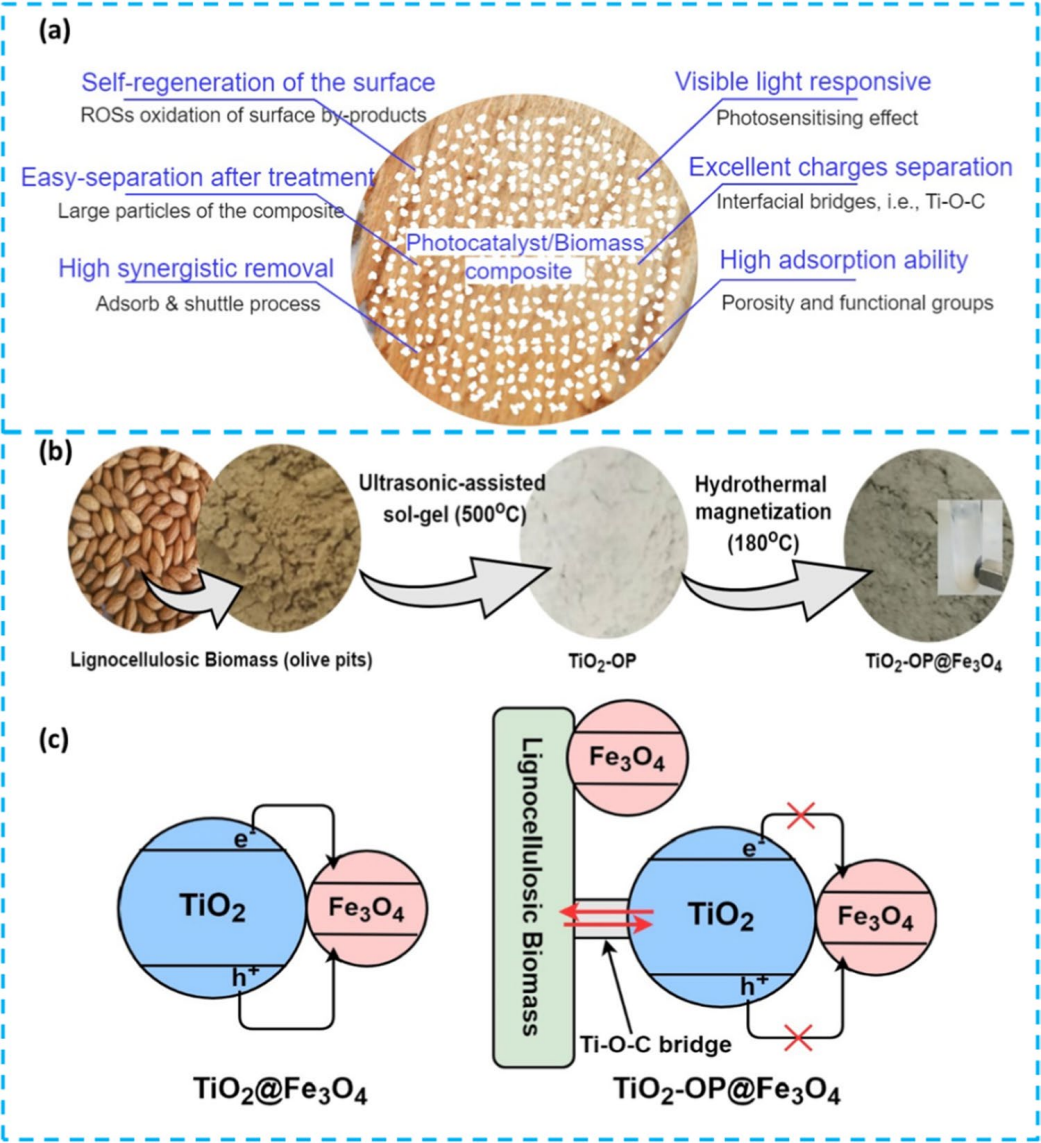
**a** Scheme shows the main benefits of combining biomass and photocatalytic nanoparticles. **b** Synthesis process of magnetic TiO_2_-biomass for water purification. **c** Photocatalytic mechanism on the surface of TiO_2_/Fe_3_O_4_ and TiO_2_/Fe_3_O_4_@biomass composites; **b** and **c** were reproduced from Djellabi et al. (2019a, b, c, d); copyright 2023 Elsevier

Lignocellulosic biomass–based materials have been combined with many photocatalytic active materials, displaying synergistic effect between adsorption and photocatalysis. Indeed, carbon materials have been regarded as successful supports for photocatalysts because of their structure and physical stability (Baek et al. 2013; How et al. 2014; Mian and Liu 2018; Zhang et al. 2018a, b). Several mechanism pathways and eco-benefits can be obtained when the photocatalytic nanoparticles and lignocellulosic biomass are combined (Fig. 12a), as follows:

- *Adsorb and shuttle process*: In such kinds of materials, a systematic cycling process of adsorption followed by photooxidation occurs. The sorption area concentrates the organic pollutants near the photoactive area for enhanced ROS attack (Djellabi et al. 2020a, b, c, Saber et al. 2021; Abderrahim et al. 2022b). In naked-based photocatalysts, the photocatalytic kinetics is very slow because of low adsorption ability, and as a consequence, most of produced ROSs will not be very interactive with organic pollutants (Ollis 2018, Wang, Zhang et al. 2020a, b). If we use a naked photocatalyst with high adsorptive ability, the surface of the photocatalyst would be covered, and no further light penetration and photocatalytic activity would occur. In a system where a photoactive area exists on an adsorptive area, the concentration of pollutants would not affect the photoactivity of uncoated photoactive nanoparticles.
- *Enhanced visible light response*: The hybridization of photocatalysts with carbon-based materials leads to improved photoactivity under visible light through different mechanisms, including (i) photosensitizing, where the biomass polymer could absorb the light and transfer its energy to the photocatalyst, and (ii) decrease in band gap because of the surface interaction during the synthesis, such as the formation of bridge bonds. Djellabi et al. proved the formation of Ti–O–C in TiO_2_/biomass-based composite (Djellabi et al. 2019a, b, c, d). In addition to the enhanced light absorption, an excellent charges separation would occur through this bridge (Zhang et al. 2020a, b).
- *Enhanced adsorption ability*: As discussed above, the adsorb and shuttle process is the primary mechanism for the oxidation of organic pollutants; the sorption ability also helps decrease the number of contaminants in water and contributes to fixing the generated toxic by-products (Djellabi et al. 2021a, b, c).
- *Self-generation of the materials*: In general, adsorption suffers from the fast deactivation of the adsorbent because if there is an accumulation of pollutants on the surface. However, in photocatalytic/adsorptive composites, the photocatalytic action plays the role of surface cleaning, wherein ROSs decrease the amount of the contaminants on the surface, allowing a longer lifetime of the photocatalyst.
- *Easy handling and recovery*: Naked photocatalytic nanoparticles are difficult to handle because of the nanosized particles. After the water treatment, it is tough to recover the particles from water, and a membrane separation step is required, increasing the process cost (Friehs et al. 2016; Djellabi et al. 2021a, b, c). However, the recovery and handling of NPs/biomass composites are easier through the simple decantation and filtration.

Several biomass-supported photoactive composites have been developed via several synthesis methods using different raw biomass supports and semiconductors. TiO_2_ hybridized with biomass-based materials has received the most attention (Kim and Kan 2016; Khataee et al. 2017; Zhang et al. 2017; Djellabi et al. 2019a, b, c, d; Lu et al. 2019; Fazal et al. 2020). Djellabi et al. (2019a, b, c, d) prepared magnetic TiO_2_-Fe_3_O_4_@biomass-based composite to oxidize organic pollutants in water. The synthesis process is shown in Fig. 12b. The authors explained the role of biomass in TiO_2_-Fe_3_O_4_@biomass as compared to bare TiO_2_-Fe_3_O_4_. Based on many reports, TiO_2_-Fe_3_O_4_ does not build a successful heterojunction due to the intense recombination of electrons/holes charges, wherein Fe_3_O_4_ plays the role of a recombination center (Beydoun et al. 2000; Abramson et al. 2009; Salamat et al. 2017). Zielińska-Jurek et al. reported the dissolution of Fe_3_O_4_ because of the electrons coming from the conduction band of the TiO_2_. To isolate TiO_2_ and Fe_3_O_4_, biomass was used to support both TiO_2_ and Fe_3_O_4_; meanwhile, biomass can play the role of electron acceptor via the Ti–O–C bridge, avoiding the transfer of dissolution of Fe_3_O_4_ (Fig. 12c). TiO_2_-Fe_3_O_4_@biomass can be quickly recovered by the magnetic field. Several studies on g-C_3_N_4_ supported on biomass materials were previously reported (Pi et al. 2015; Jeon et al. 2017; Kumar et al. 2017, 2018). Sun et al. said that C_3_N_4_/ferrite/biochar, on top of its photoactivity, can initiate Fenton or/and photo-Fenton reactions further to boost the oxidation of organic pollutants (Sun et al. 2020). Zhou et al. reported porous lignocellulose-derived *Juncus effusus* fiber–supported photocatalysts for efficient wastewater treatment (Zhou et al. 2022). The exquisite 3D microstructure of lignocellulose-derived *Juncus effusus* fiber and affluent •OH group enable it to support some catalyst powders such as g-C_3_N_4_. The lignocellulose-derived fiber-supported photocatalysts showed excellent photodegradation performance for different organic pollutants, such as organic dyes and antibiotics. Roa et al. (2021) clarified the photodegradation mechanism of lignocellulose-based material via the dye removal tests and pointed out that most of these lignocellulose-derived carbon materials possess rich pore structures and surface functional groups, which make them to show strong enrichment capacity for pollutants as base materials, making up for the inability of many powder photocatalysts to quickly capture pollutants. The enhanced structural property and presence abundant functional group greatly improved the ability of photocatalytic degradation of pollutants. ZnO/biomass-based composites for the oxidation of different organic pollutants have also been widely reported (Leichtweis et al. 2020; Yu et al. 2021; Cai et al. 2022). A decrease in the band gap in ZnO/biochar composites was reported by Gonçalves et al. (2020). The authors suggested that the reduction of e^_^/h^+^ recombination at ZnO/biochar composite; also, the sample calcined at 400 °C showed the highest production of ROSs. Other photocatalysts were coated as well on biomass-based materials. For example, BiOBr and BiOCl combined with biochar lead to improve visible light responsiveness, as discussed by Li et al. (2016a, b). Chen et al. developed magnetically recoverable biochar@ZnFe_2_O_4_/BiOBr Z-scheme by solvothermal, and the authors report that BiOBr boosts the separation of charges and the adsorption.

## Photocatalytic removal of heavy metals from water

**Fig. 13:**
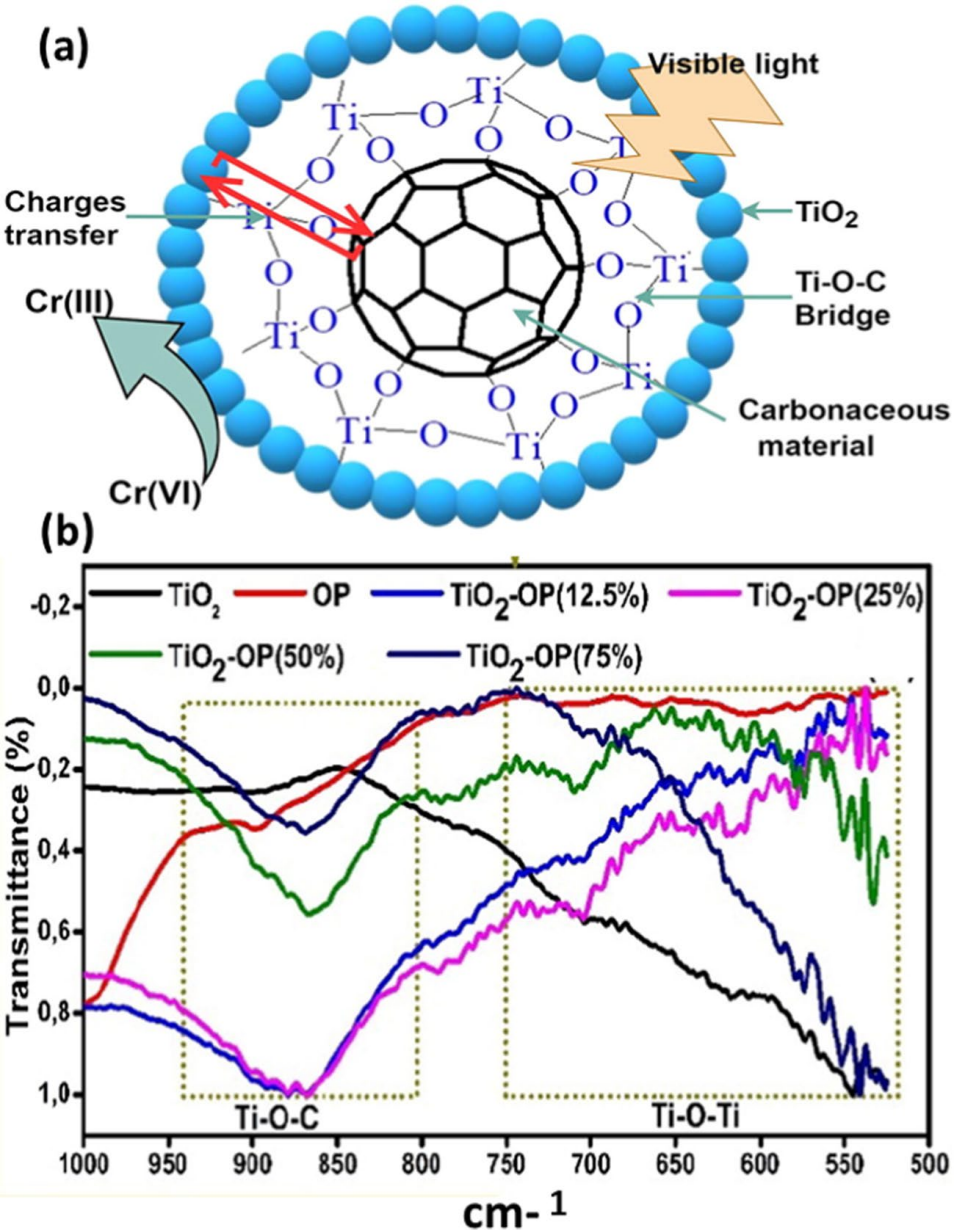
**a** Ti–O–C bridge formed between TiO_2_ nanoparticles and carbonaceous materials, reproduced with permission from Djellabi et al. (2019a, b, c, d). FTIR spectra of TiO_2_-biomass samples (OP: olive pit–based biomass), reproduced with permission from Djellabi et al. (2019a, b, c, d); copyright 2023 Elsevier

The photocatalytic reduction of heavy metals by photocatalysts supported on lignocellulose biomass materials has been widely reported and discussed (Wang et al. 2020a, b, c, d, e, f; Bhavani et al. 2022; Lu et al. 2022). The photoproduced electrons achieve the reduction of heavy metals by photocatalytic means on the conduction band of the photocatalyst and some reducing organic agents that could be produced from the oxidation of co-existed organic molecules, i.e., CO_2_^•−^ (E^0^(CO_2_/CO_2_^•−^) ≈ − 2.0 V (Marinho et al. 2017). In addition, some ROSs, such as ^−•^O_2_, can participate in reducing some heavy metals (An et al. 2020a, b). From the environmental viewpoint, reducing heavy metals to the metallic state, or in some cases to lower oxidation states, i.e., Cr(VI) to Cr(III), could reduce their toxicity and mobility in water. In addition, during the photocatalytic reduction, photodeposition occurs at the surface of the photocatalyst to produced and extract metallic species (Djellabi et al. 2016). However, the photodeposition process has been regarded as inconvenient in many reports as it deactivates the surface and blackens the light penetration/excitation processes (Zhang et al. 2018a, b). It is worth mentioning that, in general, the photocatalytic reduction of heavy metals accompanies the oxidation of organic molecules as hole scavengers (Djellabi et al. 2021c). The presence of hole scavenger molecules promotes the separation of electrons on the conduction band and limits the re-oxidation of produced metallic species (reverse reaction) by ROSs, as discussed in depth previously (Djellabi and Ghorab 2015; Djellabi et al. 2020a, b, c; Bortolotto et al. 2022). Many physical and photonic mechanisms can occur in terms of the photocatalytic reduction of heavy metals by photocatalytic NPs/biomass-based composites. In terms of TiO_2_-biomass photocatalysts, Djellabi et al. investigated the mechanistic role of the interfacial link formed between the biomass and TiO_2_ nanoparticles on the light absorption, band gap, and photocatalytic efficiency for Cr(VI) reduction (Djellabi et al. 2019a, b, c, d). It was claimed that the increase of biomass ratio in Biomass/TiO_2_ composites leads to decreased band gap due to stronger interfacial interactions. The authors suggested the formation of a Ti–O–C bridge between TiO_2_ and biomass, as shown in Fig. 13a. Employing FTIR analysis (Fig. 13b), a new peak at 850 cm^−1^ was produced when TiO_2_ and biomass were hybridized, and the intensity of the peak is related to biomass content. On top of the decrease in the band gap, enhanced charge transfer and photosensitising could produce more redox species through this bridge. Magnetic biomass-TiO_2_ for Cr(VI) reduction was also reported, wherein the co-presence of TiO_2_ and magnetic Fe_3_O_4_ boosts the photoreduction of Cr(VI) synergistically under solar light. Several photocatalytic materials have been combined with lignocellulosic biomass-based materials such as g-C_3_N_4_ (Li et al. 2022a, b), BiVO_4_ (Li et al. 2022a, b), Ag_3_PO_4_ (Abderrahim, Djellabi et al. 2022a, b), Bi_2_WO_6_ (Wang et al. 2020a, b, c, d, e, f), ZnO (Cruz et al. 2020), and photoactive polymers (Djellabi et al. 2020a, b, c; Djellabi et al. 2021a, b, c). On the other hand, the toxicity of some heavy metals can be reduced by their reduction to lower oxidation states, such as in the case of arsenic. In this regard, the oxidation by photocatalytic means is similar to the photooxidation of organic pollutants based on ROSs attack. Yan et al. reported that As(III) could be effectively removed by NiS/NiSe/3D porous biochar through a synergistic adsorption-photocatalytic process (Yan et al. 2021). The adsorbed As(III) can be photocatalytically converted into As(V) by photogenerated ROSs on the surface of biochar. Xue et al. studied the removal of As(III) by SnS_2_-supported biochar and decorated with heteropoly acid (Xue et al. 2021). The addition of phosphotungstic acid to biochar/SnS_2_ system improves the removal of As(III) by 1.5 times. TiO_2_-biochar was also used to remove As(III) by adsorption-photocatalytic system, wherein ^−•^O_2_ species were the most responsible oxidative ROSs for the oxidation of adsorbed As(III).

Similar to the photooxidation of organic pollutants, the photocatalytic removal of heavy metals by biomass supported photocatalysts materials is released through the so-called adsorb and shuttle process, wherein the highly adsorbing domain concentrates metallic species nearby the photocatalytic domain to be reduced/oxidized as discussed in depth previously (Djellabi et al. 2020a, b, c, Saber et al. 2021, Fellah et al. 2022).

## Conclusions and challenges

This review summarizes the main applications of valorized lignocellulosic biomass for environmental remediation. Lignocellulose biomass wastes can be converted into activated carbon via several ways and activation processes. The use of some techniques, i.e., microwave carbonization, might lead to obtaining highly activated carbon, having excellent porosity and functionality for enhanced adsorption of water and air pollutants. In addition, the incorporation of photocatalytic nanoparticles on the surface of lignocellulose biomass-based materials aims to solve several challenges associated with the conventional photocatalysis, such as a significant enhancement in the kinetics due to the adsorb and shuttle process, reduction in toxic-by products generation because of the highly adsorptive ability, and the improvement of light absorption and charges separation (interfacial interaction, i.e., C–O–Ti bridge, and photosensitizing). The separation of photocatalyst/carbon composites is quite easier than the recovery of nanosized naked photocatalysts. In addition, the cost of photocatalysts/lignocellulose biomass would be cheaper. From the eco-environmental point of view, the valorization of lignocellulosic biomass into carbon materials to be used as adsorbents for water and air pollutants, or to support photocatalytic particles suits very well the concepts of circular bioeconomy in terms of waste recycling, use bio-resource and sustainability. To better scale up lignocellulose biomass waste for large environmental remediation applications, some challenges need to be addressed. In terms of activated carbon from lignocellulosic biomass, sustainable approaches for the activation and carbonization with the use of harmful chemicals are required to be investigated to avoid secondary water and air pollution, e.g., the production of toxic gas of pyrolysis and use of highly toxic chemical for the activation (highly corrosive agents, i.e., KOH or H_3_PO_4_). Self-activation approaches to produce carbon materials instead of chemical or physical routes require more investigation because of its sustainability and low cost.

For photocatalytic applications, certainly, most of the studies reported that synergetic effects take place to promote the removal of pollutants as compared to simple adsorption and photocatalysis primarily due to the *adsorb and shuttle process*. In addition, the oxidation of organic contaminants on the surface of adsorbents is regarded as a self-regeneration, allowing a more prolonged use of the material. However, up to date, this approach is not considered at large scale because of other technical issues, such as the type of irradiation and photoreactor that allows better light penetration if the black photocatalyst/carbon material is used. Self-oxidation of biomass by photoproduced ROSs should be estimated. Since ROSs could oxidize the biomass, it is highly recommended to check the release of products from the biomass during photocatalytic experiments and the stability of the structure by some surface characterization. Further studies to test adsorbents and photocatalysts should be conducted in real conditions and pilot scale. Techno-economic and sustainability factors should be estimated.

Standardized protocols for carbon materials fabrication from lignocellulosic biomass should be developed based on the type of raw materials and the targeted application. In fact, even though chemical activation leads to highly active activated materials, it would be better to seek for advanced green approaches to design activated carbon–based materials in order to avoid the over usage of harmful and toxic chemicals. Most of research studies have been carried out on the valorization of a selected biomass waste; however, real municipal biomass wastes contain a variety of biomass materials. At a large scale, it is hard to separate different biomass wastes from each other. Therefore, it would be interesting to investigate the conversion of real municipal biomass wastes as a total into carbon materials to widen the valorization of real biomass wastes in to valuable products.

## Data Availability

The authors confirm that the data supporting the findings of this study are available within the article.
